# Dysferlin and the Regulation of Ca^2+^ Release in Skeletal Muscle

**DOI:** 10.3390/cells14211724

**Published:** 2025-11-03

**Authors:** Robert J. Bloch, Joaquin Muriel, Valeriy Lukyanenko

**Affiliations:** Department of Pharmacology and Physiology, School of Medicine, University of Maryland, 655 W. Baltimore St., Baltimore, MD 21201, USA

**Keywords:** excitation-contraction coupling, membrane repair, sarcolemma, RyR, couplon

## Abstract

Dysferlin is a large transmembrane protein that is mutated or absent in Limb Girdle Muscular Dystrophy Type R2 (LGMD R2). Although it may have several functions in healthy skeletal muscle, most research on dysferlin has addressed its roles in repair of the sarcolemma and in maintaining proper control of Ca^2+^ homeostasis at the triad junction, where it concentrates. Here, we review the literature on the role of dysferlin in both membrane repair and in Ca^2+^ homeostasis, with a focus on the latter. We propose that pathophysiology in LGMD R2 is in part the result of increased leak of Ca^2+^ at the triad junction, which in turn reduces the amplitude of Ca^2+^ transients and, by activating Ca^2+^-induced Ca^2+^ release, or CICR, at the triad junction, induces Ca^2+^ waves. We discuss the mechanisms that regulate Ca^2+^ leak and Ca^2+^ levels at the triad junction under physiological and pathophysiological conditions. Our results suggest that suppression of abnormal leak and CICR may be therapeutic for LGMD R2 and other diseases of muscle linked to dysregulation of Ca^2+^ homeostasis.

## 1. Introduction

Dysferlin has been linked to Limb Girdle Muscular Dystrophy type R2 (LGMD R2), which encompasses several phenotypes previously referred to as Miyoshi myopathy (MMD1), LGMD 2B, distal myopathy of the anterior tibialis (DMAT), and asymptomatic hyperCKemia. These diseases are typically autosomal recessive (but see: [1]) and most frequently manifest in young adulthood, leading to wheelchair use within 10–15 years ([2,3,4]). The *DYSF* gene, which encodes dysferlin, is located on chromosome 2p13.2. It is comprised of 58 exons that are transcribed into an mRNA of ~6900 bp, which is translated into a protein of ~230 kDa. Several splice variants of dysferlin have been identified, with the 2080 amino acid form (GI: 20137708; GI: 3600028) predominating in skeletal muscle [5]. Muscle biopsies from individuals with LGMD R2 have little to no dysferlin, suggesting that the pathogenic mutations destabilize dysferlin mRNA or result in reduced synthesis or premature degradation of the protein [5,6]. Although cardiac function is relatively spared [3,7,8,9] and lifespan is usually not affected, dysferlinopathies can severely impact quality of life [4,10].

**Figure 1:**
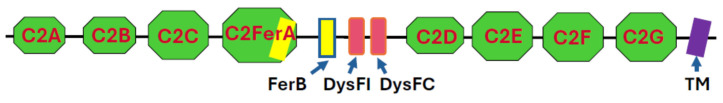
Domain structure of dysferlin. C2 domains are in green, Fer domains in yellow, Dysf domains in red, and the transmembrane (TM) domain in purple. With the exception of a short extracellular sequence, depicted to the right of the TM domain, the protein is exposed to the cytoplasm. Not drawn to scale.

Like other members of the ferlin superfamily [11,12,13,14], dysferlin is a type II transmembrane protein comprised of C2 and ferlin (FerA and FerB) domains. It also contains two dysferlin-specific domains (DysFI and DysFC) in the approximate middle of its sequence, flanked by the C2 domains (Figure 1). The structures and relationships among these domains were first modeled with RoseTTAFold [15] and, more recently, revealed by cryo-EM [16,17].

The seven C2 domains that were identified originally are termed C2A through C2G, starting from the N-terminus. C2A, C2B, and C2D are typical C2 domains, primarily β-sheet structures with masses of ~14 kDa. C2C, C2E, C2F, and C2G also contain α-helical elements and are larger (21–25 kDa). A more recently identified domain, termed C2-FerA [15], has a molecular mass of ~31 kDa and is just C-terminal to C2C. It is so named because it has a FerA domain, primarily α-helical in structure, appended to its β-sheet fold. Cryo-EM studies reveal that all the C2, Fer, and Dysf domains except C2A form an elongated, irregular ovoid structure, with the C2G domain protruding towards the membrane surface [16,17]. When bound to a lipid bilayer in the presence of Ca^2+^, the association of C2G with C2B drives the formation of a ring-like structure [17]. The C2A domain’s structure has been predicted [15] but not resolved by cryo-EM [16,17], presumably because of its high mobility with respect to the protein core, to which it is linked by a ~100 residue polypeptide. The protein can extend more than 20 nm into the cytoplasm from the membrane surface [16], depending on the orientation of the cytoplasmic domain with respect to the membrane bilayer. The transmembrane (TM; for a list of abbreviations used in this paper, please see Table 1) sequence of dysferlin is 22 amino acids in length and so is likely to be tilted with respect to the plane of the lipid bilayer [15]. Fewer than 15 amino acid residues are exposed on the extracellular surface of the membrane.

Native gel electrophoresis of the protein, expressed by baculovirus in Sf9 cells, suggests that the bulk of the protein forms homodimers [16], in agreement with earlier, fluorescence correlation spectroscopy studies of a fluorescently tagged version of the protein [18]. Surprisingly, cryo-EM suggests that the dimer is asymmetric, with the C2B, C2D, C2E, and DysFI domains of one monomer contacting the C2B, C2C, C2FerA, FerB, and DysFC domains of the second [16]. It remains to be determined if this is the predominant form in muscle, and if the monomer, the homodimer, or larger homo-oligomers, which are also apparent in the native gels [16], is the active form of the protein in situ.

Since its initial discovery and linkage to diseases of skeletal muscle, dysferlin’s role in maintaining muscle health has been intensively investigated. Potential roles in vesicle trafficking [12,14,19,20,21], regulation of oxidative and cellular metabolism [22,23,24,25,26], ROS generation [27,28,29,30], lipid accumulation [23,31,32,33], and myogenesis [34,35,36,37,38,39] have been well documented, primarily by comparing wild-type muscle to muscle from dysferlin-null mice. Dysferlin-null muscle also undergoes inflammation, which is likely to contribute to the dysferlinopathic phenotype [40,41,42,43,44,45,46,47,48]. Here, we focus on the roles of dysferlin in the regulation of Ca^2+^ signaling and in sarcolemmal repair.

## 2. Dysferlin and Sarcolemmal Repair

A role for dysferlin in membrane repair [49] (reviewed in [9,19,20,49,50,51,52,53,54]) has been supported by a range of experiments. Immunofluorescence imaging of sections of unfixed skeletal muscle suggested that dysferlin was present primarily at the sarcolemma of healthy skeletal myofibers, sometimes accompanied by numerous small puncta, presumed to be vesicles, in the myoplasm [36,41,49,55,56,57,58,59,60,61,62,63]. Ultrastructural studies showed membrane-bound vesicles accumulated under the sarcolemma in dysferlin-null but not control fibers [39,64,65], consistent with a defect in the fusion of vesicles with the plasma membrane, which was assumed to be responsible for repairing the sarcolemma after injury [19,20] (Other ultrastructural defects were also present, however: see [64,66]).

Evidence to support this hypothesis first came from in vitro studies of the sarcolemmae of myofibers that were wounded by a laser beam in the presence of a dye that fluoresces when it associates with the lipid bilayer. This method, which had previously been used successfully with studies of membrane resealing in sea urchin eggs [67,68,69], showed that dye uptake was significantly enhanced in myofibers from dysferlin-null mice [49,70,71,72,73,74,75]. Rapid and complete membrane repair in healthy fibers depended on the presence of Ca^2+^ [49,70,71,76,77,78,79,80], consistent with the requirement for Ca^2+^ in membrane fusion events, though the source of the Ca^2+^ ions could be either intracellular or extracellular. Furthermore, dysferlin concentrated at the repair site after laser wounding [76], has both phospholipid membrane and Ca^2+^ binding activities [17,81,82,83,84,85,86,87], and can promote membrane fusion [49,58,70,88] (reviewed in [9,12,19,20,89]). The homology of dysferlin’s most N-terminal C2 domain, C2A, to synaptotagmin 1 (SYT1: homology of identical and conserved residues = 47%), which shares the ability to bind both Ca^2+^ and phospholipids with dysferlin’s C2A domain [90,91], and which mediates the fusion of synaptic vesicles with the presynaptic membrane in neurons (reviewed in [92,93]), appeared to make this accounting of dysferlin’s function complete.

This idea is consistent with the fact that a number of other proteins that accumulate with dysferlin at sites of sarcolemmal repair, and that may play key roles in the process (reviewed in [20,50,51,52,94]) have also been identified following laser wounding. These include mitsugumin 53 (MG53, also known as TRIM72) [71,95], caveolin 3 [95], and annexins [60,76,96,97,98,99], as well as the actin cytoskeleton [70,75]. Notably, MG53, caveolin 3, and annexins associate with dysferlin [58,88,100,101,102], and they, together with actin, are linked to diseases of skeletal muscle [99,103,104,105,106,107,108].

The model for repair of the sarcolemma that was proposed on the basis of these and other studies invoked fusion events between cytoplasmic vesicles and the sarcolemma, activated by the influx of Ca^2+^ upon injury from the extracellular milieu or from intracellular stores, and postulated to be mediated by dysferlin on both membrane surfaces [19,20,54,82,89,109]. The ability of dysferlin to homodimerize [16,18] is consistent with this model. Subsequent studies suggested that the model was incomplete, as it omitted the possibly primary role that annexins play in sarcolemmal repair [58,76,94,98,99,105,110], as well as the potential roles of enzymes, such as sphingomyelinase [111] and other proteins associated with fusion events, such as syntaxin 4 and SNAP-23 [88,112], that may function in the absence of dysferlin [112]. Nevertheless, a role for dysferlin in membrane repair has remained the focus of study in many laboratories.

Results consistent with this model were found using methods other than laser wounding to challenge the integrity of the sarcolemma of dysferlin-null myofibers. In an early study, the sarcolemma was damaged with a needle, and repair was accompanied by a large, dysferlin-rich “scar” at the repair site [49]. A variant of the model was suggested by studies in which injury to the plasma membrane of myoblasts or myotubes was caused by bombardment with silica microparticles, which generated membrane-lined tunnels through the cells that became associated with a fragment of dysferlin [113]. Remarkably, this process involves cleavage of dysferlin by calpain 3, which also binds to dysferlin [94,114]. Notably, its ability to cleave dysferlin requires a specific alternatively spliced form of dysferlin containing the sequence encoded by exon 40a [115]. Cleavage by calpain 3 generates a 72 kDa synaptotagmin-like fragment that remains associated with the membrane lining the tunnel. If the 40a-inclusive isoform form is absent, or if calpain is inhibited, dysferlin fails to line the tunnel [113,115,116]. As expected for a calpain-mediated process, Ca^2+^ is required for this repair mechanism, consistent with an earlier report on membrane repair in fibroblast cells injured by scraping [117]. In biolistic injury, the source of Ca^2+^ is extracellular, and flux into the damaged region is mediated by L-type Ca^2+^ channels [113]. To date, however, the relationship of this mechanism of sarcolemmal repair to that responsible for repair after laser wounding has not been examined.

Recent experiments addressing membrane repair were performed with myobundles, formed in vitro by myoblasts isolated from healthy or dysferlin-null muscle, that were exposed to hypoosmotic solutions [25]. These experiments did not assay either dye uptake into damaged membranes or the trafficking of dysferlin to sites of damage, however, so it is difficult to know if the damage sustained by the myotubes in the bundles was localized to the sarcolemma, to intracellular membrane compartments like the SR, or if other cytoplasmic factors were affected by the osmotic shock. It is therefore unclear if recovery of the parameters assayed after the bundles were returned to isosmolar conditions in these experiments was due to sarcolemmal repair or to other factors (for more on the effects of hypoosmotic shock, please see below).

Subsequent studies raised questions about the relevance of dysferlin’s role in membrane repair for muscle health, however. In particular, one study examined muscle expressing a “nanodysferlin”, designed to be therapeutic [118], while another examined muscle expressing a small form of dysferlin encountered in a patient with only a mild form of dysferlinopathy [73,119]. A third study examined the effects of overexpressing myoferlin, a dysferlin homolog [11,12,13,120], on membrane repair and muscle health [73]. These experiments showed that membrane repair was not sufficient to restore normal histology or physiology. Dysferlin-null B6.A^prmd^/GeneJ (also known as BlAJ) mice that over-expressed myoferlin as a result of transgenesis supported control levels of membrane repair after laser wounding but still showed histopathology [73]. BlAJ muscles transduced to express the patient-derived minidysferlin also supported membrane repair [119], but they failed to show significant improvements in muscle histology and, moreover, were not protected against the effects of eccentric injury [73], unlike control, dysferlin-positive mice [45,121,122]. BlAJ mice treated with AAV to express a “nanodysferlin” containing the C2A, C2B, C2C, FerA, DysF, and C2G domains also showed substantial membrane repair after laser wounding, but they only partially suppressed histopathology [118]. These observations suggest that, although dysferlin likely plays a role in membrane repair, its role as a repair protein is not sufficient for muscle health.

Questions about the relevance of the in vitro injury by laser wounding, widely used to investigate dysferlin’s role in membrane repair, also arose from experiments in which wild-type and dysferlin-null A/J mice were exposed to a more physiological injury, i.e., repeated high-strain eccentric contractions in vivo. These experiments, based on earlier studies of rats subjected to either high- or low-strain injuries [123], utilized 3-month-old A/J mice as the dysferlin-null murine model. This strain, unlike several other strains of dysferlin-null mice that have been studied (SJL/J, B10.SJL-Dysf^im^/AwaJ, BlAJ, B6.129S6-*Dysf^tm2.^^1Kcam^*/*J*), does not exhibit obvious signs of histopathology or loss of muscle function, which could complicate interpretation of the results. A/J mice subjected to injury by repeated high-strain eccentric contractions initially lose about the same amount of force as their wild-type counterparts, but they recover more slowly. Wild-type mice recover full activity within three days in a process independent of new myofiber formation, whereas A/J mice need two weeks to recover in a process that requires regeneration [45,46,121]. In this regard, A/J mice may be an unique dysferlin-null model, as skeletal muscles in other dysferlin-null strains that, unlike A/J, show histopathology and are undergoing muscle degeneration and regeneration, recover more rapidly after eccentric injury [122], perhaps because regeneration in those strains is an ongoing process. Remarkably, however, when the injury is performed in the presence of fluorescent dextran, injected intraperitoneally, the same number of A/J and control myofibers take up the dye and retain it equally well for three days [45]. Subsequent loss of the dye from A/J fibers is due to fiber death, which is associated with delayed but massive inflammation and their ultimate replacement by newly formed fibers [45,46,121]. Furthermore, protection against injury in vivo is afforded by diltiazem [124], a blocker of the L-type Ca^2+^ channel (LTCC). This suggests that Ca^2+^ is required for the damage caused by eccentric contractions. Overall, these results show that, although dysferlin-null muscle experiences fiber loss after several days that requires an initial influx of Ca^2+^, it can effectively reseal its sarcolemma immediately after a pathophysiological injury in vivo.

We believe that the apparently paradoxical results of studies of eccentrically injured muscle and those from myofibers injured by laser wounding are consistent, however. Laser wounding typically uses laser beams of ~5 µm in diameter, which likely damage not only the sarcolemma but also the nearby transverse tubules, rendering them more exposed to the lipophilic dye used to track damage. As the transverse tubules of dysferlin-null muscle are more susceptible to damage than wild-type muscle [70,124,125], greater uptake would be expected. Moreover, although the amount of membrane that takes up dye after laser wounding is greater in dysferlin-null myofibers than in controls, the dye does not gain access to all the intracellular membranes of the wounded fibers—which it should be able to do if repair had failed entirely—and dye uptake reaches a plateau over approximately the same time course as the controls (though the plateau level is considerably higher). These results suggest that, although dysferlin plays a significant role in limiting the extent of reorganization that membranes experience following laser wounding, it is not necessary for membrane repair, which is consistent with our results with A/J muscles subjected to eccentric contractions.

If this is the case, might dysferlin play additional roles that are critical for muscle health? To investigate this question, we developed new methods to study the distribution of dysferlin in skeletal muscle and then to examine its role in a key activity that underpins muscle health—the regulation of Ca^2+^ homeostasis and signaling.

## 3. Dysferlin Is a Transverse Tubule Protein That Concentrates at Triad Junctions

As mentioned above, standard immunofluorescence studies localized dysferlin to the sarcolemma and, in some cases, to many punctae in the myoplasm, which could be interpreted to be vesicles [42,49,56,57,58,59,60,61,62,63,101,126]. These experiments were routinely performed with unfixed muscle tissue that was snap-frozen and cryosectioned. We revisited this result by studying paraformaldehyde-fixed and snap-frozen muscles that we cryosectioned and then exposed the cryosections to “antigen unmasking” [62]. This procedure involved heating the sections in a mild acidic solution at 90 °C for 10 min and then returning them to buffered saline at ambient temperature. Immunolabeling of cross sections processed in this way showed dysferlin distributed throughout the myoplasm in a reticulum, with very little labeling at the sarcolemma. (NB: We use the term “sarcolemma” to indicate the outer plasma membrane of the muscle fiber. It does not include the transverse tubules (TT), which we consider to be a distinct membrane compartment of striated muscle). This pattern could be explained if dysferlin is primarily in the sarcoplasmic reticulum (SR) or in the TT, both of which appear reticular in cross sections.

Immunolabeling of longitudinal sections that were also “unmasked” showed dysferlin localized primarily in double lines of punctae that flanked the Z-lines of each sarcomere [62]. Punctae with this distribution are highly likely to be triad junctions, formed where ryanodine receptors (RyR1) in the terminal cisternae of the SR come into close contact with the L-type Ca^2+^ channels (LTCC; also referred to as dihydropyridine receptors, or DHPR) in the TT. Thus, these results suggest that, rather than localizing at the sarcolemma and in intracellular vesicles, dysferlin is primarily an integral membrane component of the SR or the TT that concentrates at triad junctions.

To distinguish between these two membranes, we tagged dysferlin at its N-terminus or its C-terminus with a pH-sensitive form of GFP, pHluorin. If pHluorin is exposed to a lower pH, its fluorescence intensity diminishes. When we introduced the two tagged forms of dysferlin into skeletal myofibers via electroporation, following standard procedures [127,128], placed the transfected myofibers into tissue culture, and observed the distribution of the fluorescently tagged dysferlin, each construct appeared primarily in puncta parallel to the Z-lines of each sarcomere [124], as we observed with immunolabeling of dysferlin in longitudinal sections [62]. This suggested that the distribution we observed after antigen unmasking was correct. When we placed myofibers with dysferlin with pHluorin at its C-terminus into a more acidic medium, the fluorescence diminished. By contrast, acidification produced little change in the fluorescence of myofibers expressing dysferlin with pHluorin at its N-terminal, cytoplasmic end [124]. As the lumen of the TT, but not of the SR, is exposed to the extracellular milieu, this result clearly identifies dysferlin as a TT protein with its C-terminus exposed in the TT lumen.

Notably, dysferlin’s association with TT has also been reported in studies of developing, regenerating and adult muscle and myofibers [37,75,129,130], in stretched skeletal muscles [131], and in the heart [7,132] (reviewed in [9]).

**Figure 2:**
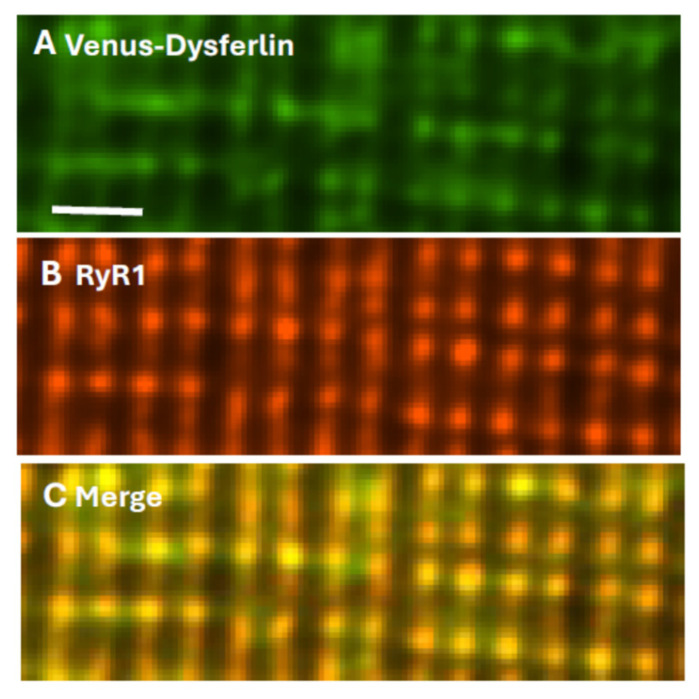
Dysferlin and RyR1 colocalize at triad junctions. Flexor digitorum brevis muscles of A/J mice were transfected by electroporation to express Venus-dysferlin (**A**), then placed in culture and immunolabeled with antibody to RyR1 (**B**). Panel (**C**) shows the merged images, with yellow indicating colocalization. Scale bar, 2 µm. Modified from ref. [124].

Final identification of dysferlin as a TT component of the triad junction (TJ) utilized myofibers transfected to express Venus-dysferlin that were subsequently placed in culture and immunolabeled with antibodies to the RyR1. The two proteins colocalize almost perfectly (Figure 2), consistent with dysferlin concentrating at TJs. Electron micrographs indicate that the TT and SR membranes are very closely apposed at TJs, with distances between the bilayers as small as 20–25 nm. As dysferlin can extend ≥25 nm from the surface of the TT [16], it has the potential to interact not only with TT proteins such as the LTCC and junctophilin, but also with the RyR1 and other proteins anchored to RyR1, including calmodulin and FKBP12.

Indeed, co-immunoprecipitation studies have identified both the LTCC and RyR1 as potential ligands of dysferlin [114,126]. Its identification as a component of the TJ raises the possibility that its role there is the same as that of other junctional proteins, i.e., to stabilize the couplons of LTCC and RyR1 to ensure physiological control of the release of Ca^2+^ in response to electrical stimulation [133,134,135,136,137].

## 4. Dysferlin, Ca^2+^ Leak and the Ca^2+^ Transient

Consistent with it serving a critical role in regulating Ca^2+^ release at the TJ, dysferlin has a marked effect on Ca^2+^ transients in A/J myofibers, compared to those from several control strains of mice. In A/J fibers, which lack dysferlin, Ca^2+^ transients have significantly smaller amplitudes than in controls [124,138,139] (see also [7]). This is due to the absence of dysferlin, and not to other genetic differences or differences in the development of the mutant fibers, as transfection of dysferlin cDNA into A/J fibers by electroporation restored the amplitude of the Ca^2+^ transient to control levels [138,139]. Moreover, when exogenous dysferlin was expressed at only one end of the myofiber, that end showed higher amplitudes [138]. Thus, the presence of dysferlin enhances the amplitude of the Ca^2+^ transient.

**Figure 3:**
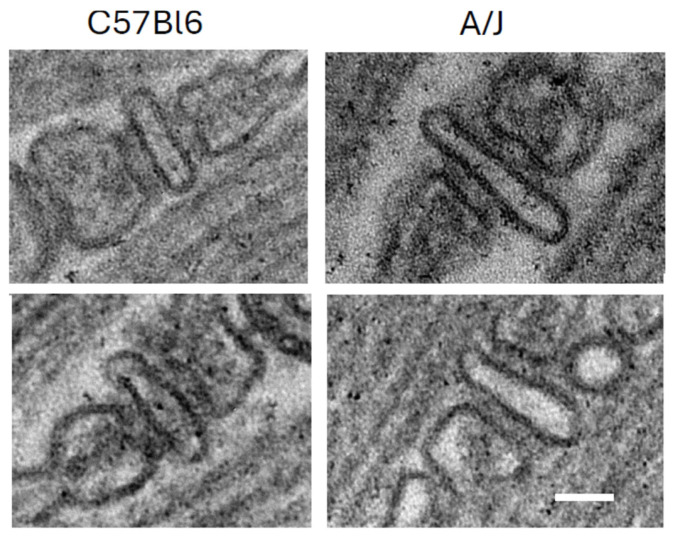
Triad junctions from wild-type (C57Bl/6) and dysferlin-null (A/J) skeletal muscle. Scale bar, 50 nm. Two examples of each are shown, with WT on the left and dysferlin-null A/J on the right.

The mechanism underlying this enhancement is not yet fully understood. The possibility that these changes can be explained by a lower density of LTCC and RyR1 in dysferlin-null muscle seems unlikely, as both proteins appear to be expressed equally well in dysferlin-null A/J and control muscle [124]. Similarly, although there are some reports that dysferlin promotes tubulation and that the TT compartment forms abnormally in dysferlin-null muscle [7,129,132], our results with A/J muscles suggest that the TT are normal [124]. They should therefore carry the action potential into the interior of muscle fibers as effectively as they do in wild-type muscles. Subtle changes, such as sites where TTs may be pinched off, are difficult to rule out with fluorescence imaging, however. Slight misalignment of the LTCC and the RyR1, which form the couplons, remains a possible reason that transient amplitudes in dysferlin-null muscle are low (e.g., [140]), although our own ultrastructural studies do not reveal any obvious defects at the TJs of A/J fibers (Figure 3).

The explanation that we propose for the lower amplitudes of the Ca^2+^ transients in dysferlin-null A/J myofibers is that the Ca^2+^ stores in the terminal cisternae of the SR are lower than in controls, due to a small but significant leak of Ca^2+^ from the SR through the RyR1 into the junctional cleft. Diminished stores would generate smaller transients. Furthermore, higher junctional [Ca^2+^] from the increased leak could also lower the driving force for Ca^2+^ movement out of the SR stores, where the [Ca^2+^]_free_ is normally ~400 µM [141,142,143,144]. This, too, would reduce the amplitude of the Ca^2+^ transient, if the [Ca^2+^]_TJ_ is high. (As discussed below, only a small increase in the number of Ca^2+^ ions in the TJ would generate a substantial increase in [Ca^2+^]_TJ_.) In addition, increases in [Ca^2+^]_TJ_ could promote additional leak through the RyR1 via Ca^2+^-induced Ca^2+^ release (CICR), to diminish the transient amplitude further. A CICR-mediated mechanism would likely be self-perpetuating unless interrupted by biochemical or pharmacological means.

Although the leak of Ca^2+^ into the TJ has not yet been measured directly, the Launikonis laboratory has developed an elegant method for detecting it indirectly [30,145]. Their studies utilize skinned muscle fibers in which the TTs are pinched off and sealed in the presence of a fluorescent Ca^2+^ indicator. When Ca^2+^ leaks into the TJ volume, it is pumped by the Na^+^,Ca^2+^-exchanger, NCX, and the plasma membrane Ca^2+^-ATPase, PMCA, into the TT lumen, where it is detected by the fluorescent indicator. Incubation of the skinned fibers in the presence of inhibitors of RyR1 reduces the Ca^2+^ signal in the TT lumen, indicating that leak through the RyR1 contributes significantly to the Ca^2+^ signal [30,145]. Notably, this occurs in resting, skinned fibers. As the process of skinning may promote leak through the RyR1 [146,147,148], there may be some challenges in concluding from these results that Ca^2+^ leak occurs in healthy muscle under resting conditions, and, if it does occur, what its absolute magnitude is. Low levels of leak, under at least partial control of the sympathetic nervous system, are highly likely to occur under physiological conditions to regulate thermogenesis, however [30,149,150,151,152,153,154].

Launikonis and his colleagues have reported further that the magnitude of the leak increases in myofibers from malignant hyperthermic muscle [155], which are known to have leaky RyR1 [156,157,158,159], as well as in myofibers from dysferlin-null mice [145]. This suggests that these diseases are “couplonopathies” [135,136] that result in excessive levels of Ca^2+^ in the TJ volume, which, as noted above, would be self-perpetuating and likely associated with pathogenesis [160].

In considering the magnitude of the leak in the absence of dysferlin, it is important to bear in mind the extremely small volume lying between the TT and the TC of the SR at triad junctions. For a region of the TJ that is ~500 nm in length and ~70 nm in width, with a membrane-to-membrane distance of ~20 nm [137], the total solution volume would be ~7 × 10^5^ nm^3^, or ~7 × 10^−24^ L, even without accounting for the volumes occupied by the junctional proteins. In the steady state, there is therefore less than one free Ca^2+^ ion in the junctional volume. Indeed, the introduction of a single free Ca^2+^ ion would raise the [Ca^2+^]_free,TJ_ above 1 µM—well above the concentration of myoplasmic [Ca^2+^]_free_ in relaxed skeletal muscle (50–100 nM; e.g., [161,162,163]).

Concentrations of Ca^2+^ in the low micromolar range should be sufficient to trigger additional Ca^2+^ release via CICR [160,164,165,166,167]. A volume of the TJ of 7 × 10^−24^ L contains ~40 RyR1 subunits [137], none of which would access free Ca^2+^ ions in the junctional volume without leak, and only a single subunit could bind that single Ca^2+^ ion if it were present. If the Ca^2+^ flux through an open RyR1 is ~10^3^/msec [168,169], only a small increase in the leakiness of a single RyR1 could raise [Ca^2+^]_TJ_ significantly, which could be more than sufficient to induce Ca^2+^ transients via CICR in the short time required (0.3 to a few msec) [164,170]. (More would of course be needed if Ca^2+^ were bound to other TJ proteins, or if the Ca^2+^ that leaks into the TJ were free to diffuse into the myoplasm, which may be unlikely, however [166,171].) Given these values, the requirements of thermogenesis, and the stresses on the TJ exerted by muscle contraction and relaxation, Ca^2+^ leak is highly likely to occur, even in healthy skeletal muscle, and thus, CICR should be more commonly observed.

CICR is rare in healthy, intact skeletal muscle, however, despite the reports from several laboratories that Ca^2+^ leak occurs [151,164,170,172,173]. This suggests either that leak does not occur at levels sufficient to stimulate CICR, which would be inconsistent with our estimates above, or that other mechanisms suppress CICR at the physiological levels of leak in healthy muscle, leaving voltage-induced release predominant [174]. The mechanisms that suppress CICR must still be identified, but drugs, ions, and small molecules that bind to the RyR1 and modulate its gating kinetics, as well as biochemical approaches leading to post-translational modifications (PTMs) of the RyR1, are likely candidates.

Small molecules, such as ruthenium red, and drugs such as ryanodine and dantrolene, inhibit RyR1 and block CICR [165]. Small decreases in cytoplasmic pH, associated with exercise, can also reduce leak [165,175]. Likewise, Mg^2+^ at concentrations of ≥50 µM is inhibitory [165,176]. As resting [Mg^2+^]_free_ is 0.3–1.5 mM [175,177,178], it may well suppress excessive Ca^2+^ leak, but it is unlikely to shut it down completely. This may be because cytoplasmic ATP concentrations, normally 5–8 mM [178,179,180,181], are high enough to blunt the inhibitory effects of Mg^2+^ [165,175].

Leak is also promoted by nitrosylation of RyR1 at cysteine residues and by PKA-mediated phosphorylation of RyR1. Several laboratories have reported that PTMs associated with ROS and nitrosylation increase the spontaneous release of Ca^2+^ through the RyR1 [182,183,184,185,186,187,188,189,190,191]. The source of the reactive NO moieties is likely to be NOX2 and NOX4 [183], both of which are expressed in skeletal muscle and are activated by exercise [192,193,194,195,196,197,198,199,200,201,202,203,204]. Exercise also promotes an increase in Ca^2+^ leak via phosphorylation of RyR1 by PKA at position S2843 [194,195] (but see [181]). Phosphorylation of this residue has been linked to skeletal muscle weakness in a wide range of myopathies [182,187,196,205,206,207,208,209] (see also [210]). Moreover, replacing S2843 with alanine, which prevents phosphorylation, may significantly reduce Ca^2+^ leak [211] (but see [181]), implicating this site specifically in the activation of leak.

Suppression of leak by these PTMs can be effected by glutathione, present at low mM concentrations in skeletal muscle, to reverse nitrosylation [212,213,214,215] (see also [216]) and by the activity of protein phosphatases, although these must still be identified. Other enzymatic modifications may also be involved.

These results strongly suggest that excessive leak is promoted by PTM of RyR1 that is linked to exercise, metabolic changes, or disease, and that targeting the underlying mechanisms may be beneficial in treating a wide range of skeletal myopathies. The focus of many of these studies has been on calstabin (also known as FKBP12) [217,218,219], a small protein that is bound to RyR1 in situ and stabilizes it in the closed state. Dissociation of calstabin occurs upon hypernitrosylation or hyperphosphorylation of RyR1, with their incumbent pathological effects [220,221,222]. If Ca^2+^ leak occurs in resting, healthy muscle, however, calstabin alone is not sufficient to suppress it completely. To our knowledge, the possibility that the low levels of leak in healthy muscle are controlled by the interactions of the RyR1 with the LTCC, or by the RyR1 molecules in the TJ that are not coupled to LTCCs [137,223], has not yet been tested.

As discussed above, the cleft of the TJ is small enough to be considered a “privileged” nanodomain. This can make immunolabeling a challenge, presumably because antibodies needed for labeling are sterically hindered from accessing their targets in the junction. NO, responsible for S-nitrosylation of RyR1, and glutathione, which would reverse this process, may have relatively free access to the junctional volume, but access of cytoplasmic enzymes, such as PKA and protein phosphatases, which would regulate the state of phosphorylation of RyR1 and perhaps other proteins of the TJ, may be limited. This raises the possibility that they are integral components of the TJ, where they could interact directly with junctional proteins, including RyR1. Small molecules may also have restricted access to the junctional volume. For example, Rhod2 (M_r_ = 791 Da) probably cannot access the junction as freely as BAPTA (M_r_ = 476 Da), a potent Ca^2+^ chelator to which it is chemically related. Remarkably, BAPTA-AM applied to A/J myofibers at 10 nM restores the amplitude of the Ca^2+^ transient to control levels, presumably by chelating Ca^2+^ in the TJ and suppressing CICR-associated decreases in Ca^2+^ stores in the terminal cisternae, whereas Rhod2-AM applied at 4 µM does not [139].

**Figure 4:**
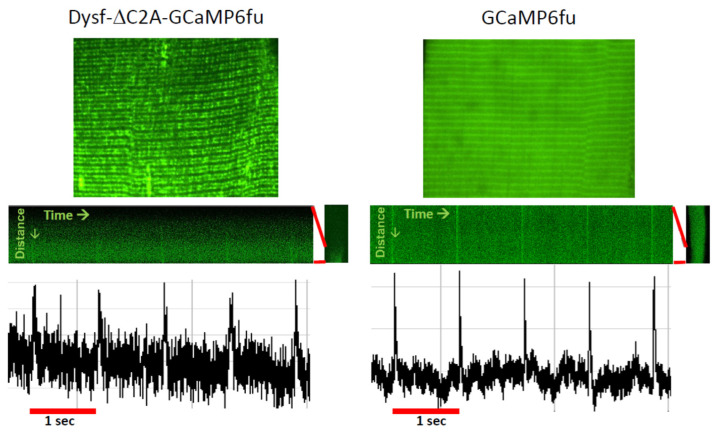
Ca^2+^ transients monitored at the TJ with dysferlin-linked GCaMP6fu. A/J myofibers were transfected to express GCaMP6fu, or dysferlin containing GCaMP6fu in place of its C2A domain. Previous results showed that this dysferlin construct concentrates at TJs and is as active as WT dysferlin in maintaining normal Ca^2+^ signaling (Lukyanenko et al., 2022 [139]). Voltage pulses at 1 Hz elicited small changes in the fluorescence of GCaMP6fu linked to Dysf-ΔC2A at the TJ (**left**), as well as of GCaMP6fu in the myoplasm (**right**). The results suggest that forms of dysferlin linked to fluorescent Ca^2+^ sensors can monitor both resting [Ca^2+^]_TJ_ and changes in [Ca^2+^]_TJ_ in response to depolarization. Please note: due to differences in the settings used to capture the amplitudes in the recordings of the two samples, they should not be compared directly. Based on studies in ref. [139].

This suggests that, in addition to accumulating the proteins required to form and regulate the activity of functional couplons, TJs may also be able to concentrate certain small molecules or ions and exclude others. Indeed, even Ca^2+^ may not freely diffuse into the myoplasm when it is released from the terminal cisternae of the TJ [66,171]. Measurements of the junctional Ca^2+^ concentration at rest have not yet been made, however. Experiments to address this question might be possible using fluorescent Ca^2+^ indicators of the GCaMP family linked to a variant of dysferlin that targets the TJ specifically [131]. Indeed, preliminary studies in our laboratory show that GCaMP6fu targeted to the TJ by attaching it to dysferlin lacking its C2A domain is sensitive enough to detect changes in [Ca^2+^]_TJ_ following a voltage pulse, as well as a background signal between stimuli that may report on the resting [Ca^2+^]_TJ_ (Figure 4). Given the calculation above, however, introducing an exogenous Ca^2+^ sensor like GCaMP6fu into a TJ may compromise the results.

Nevertheless, whatever the actual junctional concentrations of [Ca^2+^] may be, there is already ample evidence that the [Ca^2+^]_TJ_, rather than bulk myoplasmic [Ca^2+^], controls the amplitude of the Ca^2+^ transient.

## 5. Dysferlin, CICR, and Ca^2+^ Waves

Increased leak of Ca^2+^ into the TJ has been associated with myopathy, manifested as abnormal contractions. Malignant hyperthermia (MH) provides the clearest example. Most cases of MH are caused by mutations in RyR1, which cause higher rates of leak than the wild-type protein, especially when muscle is exposed to heat or local anesthetics (e.g., [141,158,160]). This can lead to even larger release events, mediated by CICR, which in adult skeletal muscle is pathophysiological [136,160]. Unless controlled, for example by dantrolene, which reduces the flux of Ca^2+^ through RyR1, MH can be fatal [224,225,226,227].

We argued above that dysferlin-null fibers exhibit higher than normal Ca^2+^ leak, but individuals with LGMD R2 do not exhibit unusually persistent or abnormal contractions. Some individuals experience fibrillations [228,229], however, which indicate abnormal Ca^2+^ release events. Based on our studies in vivo, described above, as well as in vitro studies of other myopathies [230,231,232,233,234], we predicted that mild osmotic shock of dysferlin-null myofibers would cause a higher incidence of abnormal release events, which we could monitor with a Ca^2+^-sensitive fluorescent dye. As we had linked dysferlin and many of its variants to Venus, we chose Rhod2 as the indicator. This was a fortuitous choice: as noted above, Rhod2 does not significantly affect the pathological release events that we wished to study, perhaps because its access to the TJ volume is limited. The injury protocol that we used consisted of a brief, mild exposure to a hypoosmotic solution, followed by a return to isosmotic conditions (OSI, for Osmotic Shock Injury).

When we subjected wild-type muscle fibers from different murine strains to OSI, the amplitudes of the Ca^2+^ transients returned to 70–80% of their initial value within 5 min, but were otherwise unchanged. In contrast, dysferlin-null A/J mouse fibers recovered only ~30% of their original amplitude, and ~55% of the fibers exhibited Ca^2+^ waves at frequencies of ~0.5 Hz [78,138,139]. Ca^2+^ waves are a strong indicator of the presence of CICR in myofibers and have been linked to myopathy [160]. They are much less frequent in control muscle fibers, even after OSI [78,138]. Similarly, Ca^2+^ sparks —the results of spontaneous Ca^2+^ release events [235,236,237,238] and a feature of other skeletal myopathies [231,233,234,239,240,241,242] —are much more frequent in osmotically shocked, dysferlin-null fibers than in controls [138]. Transfection of plasmid encoding wild-type dysferlin, tagged with Venus, into the fibers by electroporation led to full recovery of the transient and nearly complete suppression of waves, but only in the regions of the myofibers expressing the exogenous dysferlin and not in regions of the same fibers where the exogenous dysferlin was not expressed [138]. Mutants of dysferlin that are known to be pathogenic, such as Dysf-W52R and -V67D, failed to restore full activity, whereas polymorphic mutations, such as Dysf-V68L and Dysf-A84V, were as active as the wild-type protein (Lukyanenko et al., in preparation). These results indicate that myofibers lacking active dysferlin respond to OSI with smaller Ca^2+^ transients and spontaneous Ca^2+^ sparks and waves. These can all be explained by a model in which a significant Ca^2+^ leak at TJs depletes Ca^2+^ stores in nearby terminal cisternae and also activates CICR to initiate Ca^2+^ sparks and waves (please see our Graphical Abstract).

This leads to a clear prediction: any variant of dysferlin that suppresses Ca^2+^ waves after OSI should also maintain the amplitude of the Ca^2+^ transient at wild-type levels before OSI and restore it to wild-type levels after OSI. Conversely, any variant that does not suppress Ca^2+^ waves should also fail to maintain and restore Ca^2+^ transient amplitudes. Our data partially support this prediction. For example, dysferlin lacking its C2B domain (Dysf-ΔC2B) is as active in suppressing waves and supporting Ca^2+^ transients as wild-type dysferlin, whereas variants missing some of dysferlin’s other C2 domains fail to suppress waves and also fail to support the Ca^2+^ transient at wild-type levels, especially after OSI [78]. Similarly, the four nanodysferlins we studied are all unable to support Ca^2+^ transients at wild-type levels or to suppress waves [102].

The model proposed above requires that Ca^2+^ build-up in the TJ be directly linked to the defects in Ca^2+^ signaling in dysferlin-null muscle fibers. We tested this idea by introducing 10 nM BAPTA-AM to A/J myofibers before OSI and then measuring the amplitudes of the Ca^2+^ transients and the frequency of Ca^2+^ waves before and after OSI [139]. As noted above, BAPTA-AM, applied to myofibers at 10 nM concentrations, restored the amplitude of the Ca^2+^ transient in uninjured muscle to control levels. Remarkably, it also fully protected A/J muscle from the effects of OSI: Ca^2+^ transients had the same amplitudes as controls, and Ca^2+^ waves were almost completely suppressed. Even after being taken up and concentrated in fibers ~10-fold [139], this very low concentration of BAPTA-AM is unlikely to alter the bulk myoplasmic [Ca^2+^]_free_, considering the many proteins and organelles that buffer Ca^2+^ in muscle [243,244,245,246,247,248,249,250]. This suggests that BAPTA selectively concentrates in the TJ, thereby preventing Ca^2+^ build-up in the TJ and its downstream effects. As several other Ca^2+^ chelators fail to replicate any of the effects of BAPTA [131], Ca^2+^ localized to the TJ is likely to be common to all three pathological responses.

We tested this idea further by replacing dysferlin’s most N-terminal C2 domain, C2A, with GCaMP6fu, a fluorescent moiety that binds Ca^2+^ rapidly and with high affinity [251]. Dysferlin lacking its C2A domain (DysfΔC2A) concentrates at TJs like the full-length protein, and before OSI, it supports Ca^2+^ transients approximately equal in amplitude to those seen with wild-type dysferlin [78]. After OSI, however, the frequency of Ca^2+^ waves is somewhat elevated (though not as elevated as in the absence of dysferlin, perhaps because dysferlin’s C2C and C2E domains can also bind Ca^2+^ [81]), and the Ca^2+^ transient fails to recover [78]. We found that dysferlin carrying GCaMP6fu in place of C2A (DysfΔC2A-GCaMP6fu) concentrated at TJs like the full-length protein, supported transients with the same amplitudes as the wild-type protein, and suppressed waves [139]. (As shown in Figure 4, it also detects changes in [Ca^2+^]_TJ_ induced by voltage pulses) This strongly suggests that Ca^2+^ accumulation in the TJ cleft is responsible for diminishing the amplitude of the transient in dysferlin-null muscle and for promoting waves after injury. It further suggests that the C2A domain of dysferlin plays an important role in buffering junctional Ca^2+^ in wild-type muscle.

Several observations suggest that this simple model is inadequate, however. For example, dysferlin missing its C2A domain fails to sustain the normal amplitude of the Ca^2+^ transient after OSI, but it can still suppress most Ca^2+^ waves, suggesting that waves need not accompany a change in the transient. Dissociation of the two phenomena also occurs in dysferlin-null myofibers loaded with either EGTA-AM or Fluo4-AM, which at some concentrations restore the amplitude of the Ca^2+^ to control levels before or after OSI but fail to suppress Ca^2+^ waves [139]. This suggests that restoring control levels of Ca^2+^ in the terminal cisternae, presumably by reducing Ca^2+^-induced leak into the triad junction, is not sufficient to suppress CICR and Ca^2+^ waves. Thus, it seems to us that the link between Ca^2+^ in the triad junction and the suppression of CICR in dysferlin-null muscle is regulated by an additional mechanism. We speculate that this mechanism involves domains other than DysfC2A and PTMs like the ones discussed above, but that these PTMs suppress rather than promote Ca^2+^ leak.

## 6. Questions Remaining

Our studies, and those of others, clearly identify dysferlin as being required for the control of Ca^2+^ release in healthy muscle, and that, when missing or mutated, it results in abnormal Ca^2+^ release that is associated with myopathy. Important questions remain, however.

One question raised by our studies and others is how the defects in Ca^2+^ signaling that occur when dysferlin is mutated or absent are related to defects in membrane repair. Do the same mutations that cause defects in one cause defects in the other, or do pathogenic mutations only affect one function preferentially? Our studies of the “nanodysferlins” suggest that the two functions are not regulated identically [102], although all the domains that play a role in regulating Ca^2+^ release also play a role in membrane repair [78]. By contrast, our studies of dysferlin lacking individual C2 domains suggest that deletions that cause a 2–3-fold increase in the frequency of Ca^2+^ waves are accompanied by a ~2-fold decrease in membrane repair activity [78]. More detailed studies of pathogenic point mutants of dysferlin (see https://databases.lovd.nl/shared/genes/DYSF, accessed on 27 October 2025) (Lukyanenko et al., in preparation) may provide a definitive answer to this question. This, in turn, should reveal if defects in Ca^2+^ signaling, membrane repair, or both are pathogenic in LGMD R2.

How does dysferlin concentrate at triad junctions, and how does it carry out its functions there? Like other TT proteins, dysferlin is likely to be synthesized in the endoplasmic reticulum and then trafficked through the Golgi apparatus [252,253,254,255,256,257]. Does it traffic together with the LTCC? Once it is incorporated into the TT, does it stabilize the couplon directly, by interacting with LTCC or RyR1, or perhaps both proteins, with which it can associate [106,118], or does it do so indirectly? As a large protein with a number of structural domains that mediate protein–protein interactions [95,100,101,114,126,258,259,260], dysferlin has the potential to bind to and potentially scaffold other junctional proteins. As a homodimer [16,18], dysferlin has the inherent capacity to bind to identical pairs of ligands within the junctional cleft, crosslinking them in ways that would likely stabilize the junction. Understanding these interactions should reveal how dysferlin works to ensure the low levels of Ca^2+^ leak and the tight coupling of Ca^2+^ release to depolarization required for muscle health.

A key question is whether the defects in Ca^2+^ signaling or membrane repair are sufficient to cause myopathy, or if other changes in muscle, including defects downstream of Ca^2+^ dysregulation, are required for pathogenesis. Dysferlin has been implicated in a number of other processes, including inflammation [40,41,42,43,44,45,46,47,48,261], lipid accumulation [23,31,32,33], and myogenesis [34,35,36,37,38,39]. Moreover, the potential effects of changes in Ca^2+^ regulation are numerous and include Ca^2+^-mediated proteolysis [41,262,263,264,265,266], Ca^2+^-dependent signaling cascades [266,267,268] that can lead to changes in gene expression [269,270], and changes in mitochondrial function and Ca^2+^ uptake associated with ROS production [145,146,271,272,273,274], in addition to changes in contractile force. Are the defects in Ca^2+^ signaling that we have described sufficient to alter all of these downstream pathways, or only one or two, and which, if any, are directly linked to pathogenesis in LGMD R2?

Clinically, it will be essential to find a way to suppress the mechanisms responsible for dysferlinopathy. As noted above, finding a means of suppressing Ca^2+^ leak might be sufficient, and might also be helpful in treating other forms of muscular dystrophy that have been linked to dysregulation of Ca^2+^ homeostasis (e.g., [275,276,277,278,279,280,281,282,283]). Pharmacologically, this might be feasible using blockers of the RyR1, such as dantrolene or S107, both of which protect dysferlin-null muscle from OSI [25,138]. Membrane stabilizers that insert into the lipid bilayer, such as valmorolone, may also be beneficial [25]. The current focus, however, is on developing an adeno-associated viral (AAV) gene therapy, which has been used successfully in patients with spinal muscular atrophy. As dysferlin is too large a protein to encode in AAV, two alternate approaches have been tried.

In one, the ORF has been reduced from ~6300 to ~4000 bp by eliminating the coding regions for different sets of C2 domains. The smaller ORF is then incorporated into and expressed successfully by AAV. One such construct restored membrane repair and improved the histology of dysferlin-deficient BlAJ mice, but it did not reverse all the effects of the dysferlinopathy [118]. Our own results indicate that, although two other, similar “nanodysferlins” support membrane repair like the wild-type protein, neither supports normal Ca^2+^ signaling [102]. Although other combinations of domains may prove more effective, our finding that several domains of the dysferlin molecule are required for both normal Ca^2+^ signaling and normal membrane repair [78] suggests that finding such a combination will be challenging, if it is possible at all.

A second approach to expressing dysferlin via AAV transduction has been to incorporate overlapping halves of the dysferlin ORF into muscle fibers simultaneously. Recombination in situ then results in the production of full-length dysferlin. This approach has been validated in mice [284,285,286,287]. Its current limitation is that the levels of transduction of muscle fibers with both vectors may be too low to restore the levels of dysferlin to those needed for muscle health, which, at least in mice, are estimated to be 10–20% of wild-type levels [116]. Clinical trials of the dual vector approach are currently on hold, however (https://www.fiercebiotech.com/biotech/sarepta-ldmd-trials-all-hit-fda-hold-amid-newly-surfaced-safety-concerns, accessed on 1 August 2025).

## 7. Conclusions

Dysferlin is a large protein that plays a key role in stabilizing Ca^2+^ signaling in healthy muscle and that is also involved in sarcolemmal repair. Because it concentrates at TJs and likely acts by suppressing the local leak of Ca^2+^, dysferlin plays a key role in the mechanism of Ca^2+^ release that underlies excitation–contraction coupling in healthy skeletal muscle. In its absence, these mechanisms are compromised and muscular dystrophy ensues, probably due to dysregulation of several parallel or downstream pathways. Although we have learned a great deal about dysferlin and its role in muscle, much remains unknown. Finding therapies for LGMD R2 will likely rely on what we learn in the coming years.

Defects in Ca^2+^ signaling are a common feature of many forms of myopathy and muscular dystrophy. Thus, understanding the defects in dysferlinopathies may provide new insights into therapies that may be useful in treating a range of diseases of skeletal muscle. As muscular dystrophies are linked to mutations in dozens of different genes and together appear in ~0.1% of the human population, finding a common approach to treating a number of these diseases would be a blessing to the patients and their families.

## Figures and Tables

**Table 1:** Abbreviations.

LGMD R2	Limb Girdle Muscular Dystrophy type R2
TM	Transmembrane
BlAJ	Dysferlin-null B6.A^prmd^/GeneJ
LTCC	L-type Ca^2+^ channel
DHPR	Dihydropyridine receptor
RyR1	Skeletal muscle ryanodine receptor
TT	Transverse tubule
SR	Sarcoplasmic reticulum
TC	Terminal cistern
TJ	Triad junction
CICR	Ca^2+^-induced Ca^2+^ release
PKA	Protein kinase A
PTM	Post-translational modification
OSI	Osmotic shock injury
AAV	Adeno-associated virus

## Data Availability

No new data were created or analyzed in this study.

## References

[1] Folland C., Johnsen R., Gomez A.B., Trajanoski D., Davis M.R., Moore U., Straub V., Barresi R., Guglieri M., Hayhurst H. (2022). Identification of a novel heterozygous DYSF variant in a large family with a dominantly-inherited dysferlinopathy. Neuropathol. Appl. Neurobiol..

[2] Aoki M., Takahashi T. (2005). [Mutational and clinical features of Japanese patients with dysferlinopathy (Miyoshi myopathy and limb girdle muscular dystrophy type 2B)]. Rinsho Shinkeigaku.

[3] Belhassen I., Laroussi S., Sakka S., Rekik S., Lahkim L., Dammak M., Authier F.J., Mhiri C. (2023). Dysferlinopathy in Tunisia: Clinical spectrum, genetic background and prognostic profile. Neuromuscul. Disord..

[4] Nashi S., Polavarapu K., Bardhan M., Anjanappa R.M., Preethish-Kumar V., Vengalil S., Padmanabha H., Geetha T.S., Prathyusha P.V., Ramprasad V. (2023). Genotype-phenotype correlation and natural history study of dysferlinopathy: A single-centre experience from India. Neurogenetics.

[5] Liu J., Aoki M., Illa I., Wu C., Fardeau M., Angelini C., Serrano C., Urtizberea J.A., Hentati F., Hamida M.B. (1998). Dysferlin, a novel skeletal muscle gene, is mutated in Miyoshi myopathy and limb girdle muscular dystrophy. Nat. Genet..

[6] Cacciottolo M., Numitone G., Aurino S., Caserta I.R., Fanin M., Politano L., Minetti C., Ricci E., Piluso G., Angelini C. (2011). Muscular dystrophy with marked dysferlin deficiency is consistently caused by primary dysferlin gene mutations. Eur. J. Hum. Genet..

[7] Hofhuis J., Bersch K., Wagner S., Molina C., Fakuade F.E., Iyer L.M., Streckfuss-Bömeke K., Toischer K., Zelarayán L.C., Voigt N. (2020). Dysferlin links excitation-contraction coupling to structure and maintenance of the cardiac transverse-axial tubule system. Europace.

[8] Moore U., Fernandez-Torron R., Jacobs M., Gordish-Dressman H., Diaz-Manera J., James M.K., Mayhew A.G., Harris E., Guglieri M., Rufibach L.E. (2022). Cardiac and pulmonary findings in dysferlinopathy: A 3-year, longitudinal study. Muscle Nerve.

[9] Quinn C.J., Cartwright E.J., Trafford A.W., Dibb K.M. (2024). On the role of dysferlin in striated muscle: Membrane repair, t-tubules and Ca(2+) handling. J. Physiol..

[10] Feng X., Liu C., Xi J., Sun C., Yue D., Zhu W., Li J., Liang Z., Lu J., Luo S. (2020). The correlation of clinical evaluation with life quality and mental status in a Chinese cohort with dysferlinopathy. J. Clin. Neurosci..

[11] Bulankina A.V., Thoms S. (2020). Functions of vertebrate ferlins. Cells.

[12] Lek A., Evesson F.J., Sutton R.B., North K.N., Cooper S.T. (2012). Ferlins: Regulators of vesicle fusion for auditory neurotransmission, receptor trafficking and membrane repair. Traffic.

[13] Peulen O., Rademaker G., Anania S., Turtoi A., Bellahcène A., Castronovo V. (2019). Ferlin overview: From membrane to cancer biology. Cells.

[14] Redpath G.M., Sophocleous R.A., Turnbull L., Whitchurch C.B., Cooper S.T. (2016). Ferlins show tissue-specific expression and segregate as plasma membrane/late endosomal or trans-Golgi/recycling ferlins. Traffic.

[15] Dominguez M.J., McCord J.J., Sutton R.B. (2022). Redefining the architecture of ferlin proteins: Insights into multi-domain protein structure and function. PLoS ONE.

[16] Huang H.L., Grandinetti G., Heissler S.M., Chinthalapudi K. (2024). Cryo-EM structures of the membrane repair protein dysferlin. Nat. Commun..

[17] Cretu C., Chernev A., Szabó C.Z.K., Pena V., Urlaub H., Moser T., Preobraschenski J. (2025). Structural insights into lipid membrane binding by human ferlins. Embo J..

[18] Xu L., Pallikkuth S., Hou Z., Mignery G.A., Robia S.L., Han R. (2011). Dysferlin forms a dimer mediated by the C2 domains and the transmembrane domain in vitro and in living cells. PLoS ONE.

[19] Bansal D., Campbell K.P. (2004). Dysferlin and the plasma membrane repair in muscular dystrophy. Trends Cell Biol..

[20] Glover L., Brown R.H. (2007). Dysferlin in membrane trafficking and patch repair. Traffic.

[21] McDade J.R., Michele D.E. (2014). Membrane damage-induced vesicle-vesicle fusion of dysferlin-containing vesicles in muscle cells requires microtubules and kinesin. Hum. Mol. Genet..

[22] Vincent A.E., Rosa H.S., Alston C.L., Grady J.P., Rygiel K.A., Rocha M.C., Barresi R., Taylor R.W., Turnbull D.M. (2016). Dysferlin mutations and mitochondrial dysfunction. Neuromuscul. Disord..

[23] Haynes V.R., Keenan S.N., Bayliss J., Lloyd E.M., Meikle P.J., Grounds M.D., Watt M.J. (2019). Dysferlin deficiency alters lipid metabolism and remodels the skeletal muscle lipidome in mice. J. Lipid Res..

[24] Lloyd E.M., Pinniger G.J., Grounds M.D., Murphy R.M. (2022). Dysferlin deficiency results in myofiber-type specific differences in abundances of calcium-handling and glycogen metabolism proteins. Int. J. Mol. Sci..

[25] Khodabukus A., Prabhu N.K., Roberts T., Buldo M., Detwiler A., Fralish Z.D., Kondash M.E., Truskey G.A., Koves T.R., Bursac N. (2024). Bioengineered model of human LGMD2B skeletal muscle reveals roles of intracellular calcium overload in contractile and metabolic dysfunction in dysferlinopathy. Adv. Sci..

[26] Furrer R., Dilbaz S., Steurer S.A., Santos G., Karrer-Cardel B., Ritz D., Sinnreich M., Handschin C. (2025). Metabolic dysregulation contributes to the development of dysferlinopathy. Life Sci. Alliance.

[27] Terrill J.R., Radley-Crabb H.G., Iwasaki T., Lemckert F.A., Arthur P.G., Grounds M.D. (2013). Oxidative stress and pathology in muscular dystrophies: Focus on protein thiol oxidation and dysferlinopathies. FEBS J..

[28] Kombairaju P., Kerr J.P., Roche J.A., Pratt S.J.P., Lovering R.M., Sussan T.E., Kim J.H., Shi G., Biswal S., Ward C.W. (2014). Genetic silencing of Nrf2 enhances X-ROS in dysferlin-deficient muscle. Front. Physiol..

[29] García-Campos P., Báez-Matus X., Jara-Gutiérrez C., Paz-Araos M., Astorga C., Cea L.A., Rodríguez V., Bevilacqua J.A., Caviedes P., Cárdenas A.M. (2020). N-Acetylcysteine reduces skeletal muscles oxidative stress and improves grip strength in dysferlin-deficient Bla/J mice. Int. J. Mol. Sci..

[30] Meizoso-Huesca A., Pearce L., Barclay C.J., Launikonis B.S. (2022). Ca^2+^ leak through ryanodine receptor 1 regulates thermogenesis in resting skeletal muscle. Proc. Natl. Acad. Sci. USA.

[31] Grounds M.D., Terrill J.R., Radley-Crabb H.G., Robertson T., Papadimitriou J., Spuler S., Shavlakadze T. (2014). Lipid accumulation in dysferlin-deficient muscles. Am. J. Pathol..

[32] Sellers S.L., Milad N., White Z., Pascoe C., Chan R., Payne G.W., Seow C., Rossi F., Seidman M.A., Bernatchez P. (2018). Increased nonHDL cholesterol levels cause muscle wasting and ambulatory dysfunction in the mouse model of LGMD2B. J. Lipid Res..

[33] Agarwal A.K., Tunison K., Mitsche M.A., McDonald J.G., Garg A. (2019). Insights into lipid accumulation in skeletal muscle in dysferlin-deficient mice. J. Lipid Res..

[34] Hogarth M.W., Defour A., Lazarski C., Gallardo E., Manera J.D., Partridge T.A., Nagaraju K., Jaiswal J.K. (2019). Fibroadipogenic progenitors are responsible for muscle loss in limb girdle muscular dystrophy 2B. Nat. Commun..

[35] Anderson L.V., Davison K., Moss J.A., Young C., Cullen M.J., Walsh J., Johnson M.A., Bashir R., Britton S., Keers S. (1999). Dysferlin is a plasma membrane protein and is expressed early in human development. Hum. Mol. Genet..

[36] De Luna N., Gallardo E., Soriano M., Dominguez-Perles R., de la Torre C., Rojas-García R., García-Verdugo J.M., Illa I. (2006). Absence of dysferlin alters myogenin expression and delays human muscle differentiation “in vitro”. J. Biol. Chem..

[37] Klinge L., Laval S., Keers S., Haldane F., Straub V., Barresi R., Bushby K. (2007). From T-tubule to sarcolemma: Damage-induced dysferlin translocation in early myogenesis. FASEB J..

[38] Cohen T.V., Cohen J.E., Partridge T.A. (2012). Myogenesis in dysferlin-deficient myoblasts is inhibited by an intrinsic inflammatory response. Neuromuscul. Disord..

[39] Chernova O.N., Chekmareva I.A., Mavlikeev M.O., Yakovlev I.A., Kiyasov A.P., Deev R.V. (2022). Structural and ultrastructural changes in the skeletal muscles of dysferlin-deficient mice during postnatal ontogenesis. Ultrastruct. Pathol..

[40] Confalonieri P., Oliva L., Andreetta F., Lorenzoni R., Dassi P., Mariani E., Morandi L., Mora M., Cornelio F., Mantegazza R. (2003). Muscle inflammation and MHC class I up-regulation in muscular dystrophy with lack of dysferlin: An immunopathological study. J. Neuroimmunol..

[41] Fanin M., Pegoraro E., Matsuda-Asada C., Brown R.H., Angelini C. (2001). Calpain-3 and dysferlin protein screening in patients with limb-girdle dystrophy and myopathy. Neurology.

[42] Fanin M., Angelini C. (2002). Muscle pathology in dysferlin deficiency. Neuropathol. Appl. Neurobiol..

[43] Mariano A., Henning A., Han R. (2013). Dysferlin-deficient muscular dystrophy and innate immune activation. FEBS J..

[44] Rawat R., Cohen T.V., Ampong B., Francia D., Henriques-Pons A., Hoffman E.P., Nagaraju K. (2010). Inflammasome up-regulation and activation in dysferlin-deficient skeletal muscle. Am. J. Pathol..

[45] Roche J.A., Lovering R.M., Roche R., Ru L.W., Reed P.W., Bloch R.J. (2010). Extensive mononuclear infiltration and myogenesis characterize recovery of dysferlin-null skeletal muscle from contraction-induced injuries. Am. J. Physiol. Cell Physiol..

[46] Roche J.A., Tulapurkar M.E., Mueller A.L., van Rooijen N., Hasday J.D., Lovering R.M., Bloch R.J. (2015). Myofiber damage precedes macrophage infiltration after in vivo injury in dysferlin-deficient A/J mouse skeletal muscle. Am. J. Pathol..

[47] Tasca G., Pescatori M., Monforte M., Mirabella M., Iannaccone E., Frusciante R., Cubeddu T., Laschena F., Ottaviani P., Ricci E. (2012). Different molecular signatures in magnetic resonance imaging-staged facioscapulohumeral muscular dystrophy muscles. PLoS ONE.

[48] Urao N., Mirza R.E., Heydemann A., Garcia J., Koh T.J. (2016). Thrombospondin-1 levels correlate with macrophage activity and disease progression in dysferlin deficient mice. Neuromuscul. Disord..

[49] Bansal D., Miyake K., Vogel S.S., Groh S., Chen C.C., Williamson R., McNeil P.L., Campbell K.P. (2003). Defective membrane repair in dysferlin-deficient muscular dystrophy. Nature.

[50] Barthélémy F., Defour A., Lévy N., Krahn M., Bartoli M. (2018). Muscle cells fix breaches by orchestrating a membrane repair ballet. J. Neuromuscul. Dis..

[51] Cooper S.T., Head S.I. (2015). Membrane Injury and repair in the muscular dystrophies. Neuroscientist.

[52] Demonbreun A.R., McNally E.M. (2016). Plasma membrane repair in health and disease. Curr. Top. Membr..

[53] Doherty K.R., McNally E.M. (2003). Repairing the tears: Dysferlin in muscle membrane repair. Trends Mol. Med..

[54] Han R. (2011). Muscle membrane repair and inflammatory attack in dysferlinopathy. Skelet. Muscle.

[55] Böhm J., Leshinsky-Silver E., Vassilopoulos S., Le Gras S., Lerman-Sagie T., Ginzberg M., Jost B., Lev D., Laporte J. (2012). Samaritan myopathy, an ultimately benign congenital myopathy, is caused by a RYR1 mutation. Acta Neuropathol..

[56] Comerlato E.A., Scola R.H., Werneck L.C. (2005). Limb-girdle muscular dystrophy: An immunohistochemical diagnostic approach. Arq. Neuropsiquiatr..

[57] Gómez-Díaz B., Rosas-Vargas H., Roque-Ramírez B., Meza-Espinoza P., Ruano-Calderón L.A., Fernández-Valverde F., Escalante-Bautista D., Escobar-Cedillo R.E., Sánchez-Chapul L., Vargas-Cañas S. (2012). Immunodetection analysis of muscular dystrophies in Mexico. Muscle Nerve.

[58] Lennon N.J., Kho A., Bacskai B.J., Perlmutter S.L., Hyman B.T., Brown R.H. (2003). Dysferlin interacts with annexins A1 and A2 and mediates sarcolemmal wound-healing. J. Biol. Chem..

[59] Bohm C., Aoki M., Hayashi Y.K., Ho M.F., Arahata K., Brown R.H. (1999). Dysferlin is a surface membrane-associated protein that is absent in Miyoshi myopathy. Neurology.

[60] Meregalli M., Navarro C., Sitzia C., Farini A., Montani E., Wein N., Razini P., Beley C., Cassinelli L., Parolini D. (2013). Full-length dysferlin expression driven by engineered human dystrophic blood derived CD133+ stem cells. FEBS J..

[61] Nilsson M.I., Laureano M.L., Saeed M., Tarnopolsky M.A. (2013). Dysferlin aggregation in limb-girdle muscular dystrophy type 2B/Miyoshi Myopathy necessitates mutational screen for diagnosis [corrected]. Muscle Nerve.

[62] Roche J.A., Ru L.W., O’Neill A.M., Resneck W.G., Lovering R.M., Bloch R.J. (2011). Unmasking potential intracellular roles for dysferlin through improved immunolabeling methods. J. Histochem. Cytochem..

[63] Rosales X.Q., Gastier-Foster J.M., Lewis S., Vinod M., Thrush D.L., Astbury C., Pyatt R., Reshmi S., Sahenk Z., Mendell J.R. (2010). Novel diagnostic features of dysferlinopathies. Muscle Nerve.

[64] Cenacchi G., Fanin M., De Giorgi L.B., Angelini C. (2005). Ultrastructural changes in dysferlinopathy support defective membrane repair mechanism. J. Clin. Pathol..

[65] Ho M., Post C.M., Donahue L.R., Lidov H.G., Bronson R.T., Goolsby H., Watkins S.C., Cox G.A., Brown R.H. (2004). Disruption of muscle membrane and phenotype divergence in two novel mouse models of dysferlin deficiency. Hum. Mol. Genet..

[66] Chekmareva I.A., Bardakov S.N., Limaev I.S., Emelin A.M., Deev R.V. (2025). Ultrastructural changes of skeletal muscle tissue of patients with dysferlinopathy. Arkh Patol..

[67] McNeil P.L., Miyake K., Vogel S.S. (2003). The endomembrane requirement for cell surface repair. Proc. Natl. Acad. Sci. USA.

[68] McNeil P.L., Kirchhausen T. (2005). An emergency response team for membrane repair. Nat. Rev. Mol. Cell Biol..

[69] McNeil P.L., Vogel S.S., Miyake K., Terasaki M. (2000). Patching plasma membrane disruptions with cytoplasmic membrane. J. Cell Sci..

[70] Demonbreun A.R., Quattrocelli M., Barefield D.Y., Allen M.V., Swanson K.E., McNally E.M. (2016). An actin-dependent annexin complex mediates plasma membrane repair in muscle. J. Cell Biol..

[71] Gushchina L.V., Bhattacharya S., McElhanon K.E., Choi J.H., Manring H., Beck E.X., Alloush J., Weisleder N. (2017). Treatment with recombinant human MG53 protein increases membrane integrity in a mouse model of Limb Girdle Muscular Dystrophy 2B. Mol. Ther..

[72] Humphrey G.W., Mekhedov E., Blank P.S., de Morree A., Pekkurnaz G., Nagaraju K., Zimmerberg J. (2012). GREG cells, a dysferlin-deficient myogenic mouse cell line. Exp. Cell Res..

[73] Lostal W., Bartoli M., Roudaut C., Bourg N., Krahn M., Pryadkina M., Borel P., Suel L., Roche J.A., Stockholm D. (2012). Lack of correlation between outcomes of membrane repair assay and correction of dystrophic changes in experimental therapeutic strategy in dysferlinopathy. PLoS ONE.

[74] Marg A., Schoewel V., Timmel T., Schulze A., Shah C., Daumke O., Spuler S. (2012). Sarcolemmal repair is a slow process and includes EHD2. Traffic.

[75] McDade J.R., Archambeau A., Michele D.E. (2014). Rapid actin-cytoskeleton-dependent recruitment of plasma membrane-derived dysferlin at wounds is critical for muscle membrane repair. FASEB J..

[76] Bittel D.C., Chandra G., Tirunagri L.M.S., Deora A.B., Medikayala S., Scheffer L., Defour A., Jaiswal J.K. (2020). Annexin A2 Mediates Dysferlin Accumulation and Muscle Cell Membrane Repair. Cells.

[77] McDade J.R., Naylor M.T., Michele D.E. (2021). Sarcolemma wounding activates dynamin-dependent endocytosis in striated muscle. FEBS J..

[78] Muriel J., Lukyanenko V., Kwiatkowski T., Bhattacharya S., Garman D., Weisleder N., Bloch R.J. (2022). The C2 domains of dysferlin: Roles in membrane localization, Ca^2+^ signalling and sarcolemmal repair. J. Physiol..

[79] Covian-Nares J.F., Koushik S.V., Puhl H.L., Vogel S.S. (2010). Membrane wounding triggers ATP release and dysferlin-mediated intercellular calcium signaling. J. Cell Sci..

[80] Davenport N.R., Sonnemann K.J., Eliceiri K.W., Bement W.M. (2016). Membrane dynamics during cellular wound repair. Mol. Biol. Cell.

[81] Abdullah N., Padmanarayana M., Marty N.J., Johnson C.P. (2014). Quantitation of the calcium and membrane binding properties of the C2 domains of dysferlin. Biophys. J..

[82] Davis D.B., Doherty K.R., Delmonte A.J., McNally E.M. (2002). Calcium-sensitive phospholipid binding properties of normal and mutant ferlin C2 domains. J. Biol. Chem..

[83] Fuson K., Rice A., Mahling R., Snow A., Nayak K., Shanbhogue P., Meyer A.G., Redpath G.M., Hinderliter A., Cooper S.T. (2014). Alternate splicing of dysferlin C2A confers Ca^2+^-dependent and Ca^2+^-independent binding for membrane repair. Structure.

[84] Kwok E., Otto S.C., Khuu P., Carpenter A.P., Codding S.J., Reardon P.N., Vanegas J., Kumar T.M., Kuykendall C.J., Mehl R.A. (2023). The Dysferlin C2A Domain Binds PI(4,5)P2 and Penetrates Membranes. J. Mol. Biol..

[85] Marty N.J., Holman C.L., Abdullah N., Johnson C.P. (2013). The C2 domains of otoferlin, dysferlin, and myoferlin alter the packing of lipid bilayers. Biochemistry.

[86] Therrien C., Di Fulvio S., Pickles S., Sinnreich M. (2009). Characterization of lipid binding specificities of dysferlin C2 domains reveals novel interactions with phosphoinositides. Biochemistry.

[87] Wang Y., Tadayon R., Santamaria L., Mercier P., Forristal C.J., Shaw G.S. (2021). Calcium binds and rigidifies the dysferlin C2A domain in a tightly coupled manner. Biochem. J..

[88] Codding S.J., Marty N., Abdullah N., Johnson C.P. (2016). Dysferlin binds SNAREs (Soluble N-Ethylmaleimide-sensitive Factor (NSF) Attachment Protein Receptors) and stimulates membrane fusion in a calcium-sensitive manner. J. Biol. Chem..

[89] Han R., Campbell K.P. (2007). Dysferlin and muscle membrane repair. Curr. Opin. Cell Biol..

[90] Cheng X., Zhang X., Yu L., Xu H. (2015). Calcium signaling in membrane repair. Semin. Cell Dev. Biol..

[91] Li Z., Shaw G.S. (2023). Role of calcium-sensor proteins in cell membrane repair. Biosci. Rep..

[92] Rizo J. (2022). Molecular mechanisms underlying neurotransmitter release. Annu. Rev. Biophys..

[93] Südhof T.C., Rizo J. (2011). Synaptic vesicle exocytosis. Cold Spring Harb. Perspect. Biol..

[94] Koerdt S.N., Ashraf A.P.K., Gerke V. (2019). Annexins and plasma membrane repair. Curr. Top. Membr..

[95] Cai C., Weisleder N., Ko J.K., Komazaki S., Sunada Y., Nishi M., Takeshima H., Ma J. (2009). Membrane repair defects in muscular dystrophy are linked to altered interaction between MG53, caveolin-3, and dysferlin. J. Biol. Chem..

[96] Hernández-Deviez D.J., Howes M.T., Laval S.H., Bushby K., Hancock J.F., Parton R.G. (2008). Caveolin regulates endocytosis of the muscle repair protein, dysferlin. J. Biol. Chem..

[97] Carmeille R., Bouvet F., Tan S., Croissant C., Gounou C., Mamchaoui K., Mouly V., Brisson A.R., Bouter A. (2016). Membrane repair of human skeletal muscle cells requires Annexin-A5. Biochim. Biophys. Acta.

[98] Croissant C., Carmeille R., Brévart C., Bouter A. (2021). Annexins and membrane repair dysfunctions in muscular dystrophies. Int. J. Mol. Sci..

[99] Swaggart K.A., Demonbreun A.R., Vo A.H., Swanson K.E., Kim E.Y., Fahrenbach J.P., Holley-Cuthrell J., Eskin A., Chen Z., Squire K. (2014). Annexin A6 modifies muscular dystrophy by mediating sarcolemmal repair. Proc. Natl. Acad. Sci. USA.

[100] Drescher D.G., Drescher M.J., Selvakumar D., Annam N.P. (2023). Analysis of dysferlin direct interactions with putative repair proteins links apoptotic signaling to Ca^2+^ elevation via PDCD6 and FKBP8. Int. J. Mol. Sci..

[101] Matsuda C., Miyake K., Kameyama K., Keduka E., Takeshima H., Imamura T., Araki N., Nishino I., Hayashi Y. (2012). The C2A domain in dysferlin is important for association with MG53 (TRIM72). PLoS Curr..

[102] Muriel J., Lukyanenko V., Kwiatkowski T.A., Li Y., Bhattacharya S., Banford K.K., Garman D., Bulgart H.R., Sutton R.B., Weisleder N. (2024). Nanodysferlins support membrane repair and binding to TRIM72/MG53 but do not localize to t-tubules or stabilize Ca^2+^ signaling. Mol. Ther. Methods Clin. Dev..

[103] Aboumousa A., Hoogendijk J., Charlton R., Barresi R., Herrmann R., Voit T., Hudson J., Roberts M., Hilton-Jones D., Eagle M. (2008). Caveolinopathy--new mutations and additional symptoms. Neuromuscul. Disord..

[104] Cai C., Masumiya H., Weisleder N., Matsuda N., Nishi M., Hwang M., Ko J.K., Lin P., Thornton A., Zhao X. (2009). MG53 nucleates assembly of cell membrane repair machinery. Nat. Cell Biol..

[105] Defour A., Medikayala S., Van der Meulen J.H., Hogarth M.W., Holdreith N., Malatras A., Duddy W., Boehler J., Nagaraju K., Jaiswal J.K. (2017). Annexin A2 links poor myofiber repair with inflammation and adipogenic replacement of the injured muscle. Hum. Mol. Genet..

[106] Glyakina A.V., Galzitskaya O.V. (2022). Structural and functional analysis of actin point mutations leading to nemaline myopathy to elucidate their role in actin function. Biophys. Rev..

[107] Sonnemann K.J., Fitzsimons D.P., Patel J.R., Liu Y., Schneider M.F., Moss R.L., Ervasti J.M. (2006). Cytoplasmic gamma-actin is not required for skeletal muscle development but its absence leads to a progressive myopathy. Dev. Cell.

[108] Woodman S.E., Sotgia F., Galbiati F., Minetti C., Lisanti M.P. (2004). Caveolinopathies: Mutations in caveolin-3 cause four distinct autosomal dominant muscle diseases. Neurology.

[109] Poudel B.H., Fletcher S., Wilton S.D., Aung-Htut M. (2024). Limb Girdle Muscular Dystrophy Type 2B (LGMD2B): Diagnosis and therapeutic possibilities. Int. J. Mol. Sci..

[110] Boye T.L., Nylandsted J. (2016). Annexins in plasma membrane repair. Biol. Chem..

[111] Defour A., Van der Meulen J.H., Bhat R., Bigot A., Bashir R., Nagaraju K., Jaiswal J.K. (2014). Dysferlin regulates cell membrane repair by facilitating injury-triggered acid sphingomyelinase secretion. Cell Death Dis..

[112] Chen H.Y., Michele D.E. (2025). Syntaxin 4-enhanced plasma membrane repair is independent of dysferlin in skeletal muscle. Am. J. Physiol. Cell Physiol..

[113] Lek A., Evesson F.J., Lemckert F.A., Redpath G.M., Lueders A.K., Turnbull L., Whitchurch C.B., North K.N., Cooper S.T. (2013). Calpains, cleaved mini-dysferlinC72, and L-type channels underpin calcium-dependent muscle membrane repair. J. Neurosci..

[114] De Morrée A., Hensbergen P.J., van Haagen H.H., Dragan I., Deelder A.M., Hoen P.A.T., Frants R.R., van der Maarel S.M. (2010). Proteomic analysis of the dysferlin protein complex unveils its importance for sarcolemmal maintenance and integrity. PLoS ONE.

[115] Redpath G.M., Woolger N., Piper A.K., Lemckert F.A., Lek A., Greer P.A., North K.N., Cooper S.T. (2014). Calpain cleavage within dysferlin exon 40a releases a synaptotagmin-like module for membrane repair. Mol. Biol. Cell.

[116] Yasa J., Reed C.E., Bournazos A.M., Evesson F.J., Pang I., Graham M.E., Wark J.R., Nijagal B., Kwan K.H., Kwiatkowski T. (2023). Minimal expression of dysferlin prevents development of dysferlinopathy in dysferlin exon 40a knockout mice. Acta Neuropathol. Commun..

[117] Mellgren R.L., Zhang W., Miyake K., McNeil P.L. (2007). Calpain is required for the rapid, calcium-dependent repair of wounded plasma membrane. J. Biol. Chem..

[118] Llanga T., Nagy N., Conatser L., Dial C., Sutton R.B., Hirsch M.L. (2017). Structure-based designed nano-dysferlin significantly improves dysferlinopathy in BLA/J mice. Mol. Ther..

[119] Krahn M., Wein N., Bartoli M., Lostal W., Courrier S., Bourg-Alibert N., Nguyen K., Vial C., Streichenberger N., Labelle V. (2010). A naturally occurring human minidysferlin protein repairs sarcolemmal lesions in a mouse model of dysferlinopathy. Sci. Transl. Med..

[120] Posey A.D., Demonbreun A., McNally E.M. (2011). Ferlin proteins in myoblast fusion and muscle growth. Curr. Top. Dev. Biol..

[121] Roche J.A., Lovering R.M., Bloch R.J. (2008). Impaired recovery of dysferlin-null skeletal muscle after contraction-induced injury in vivo. Neuroreport.

[122] Roche J.A., Ru L.W., Bloch R.J. (2012). Distinct effects of contraction-induced injury in vivo on four different murine models of dysferlinopathy. J. Biomed. Biotechnol..

[123] Lovering R.M., Roche J.A., Bloch R.J., De Deyne P.G. (2007). Recovery of function in skeletal muscle following 2 different contraction-induced injuries. Arch. Phys. Med. Rehabil..

[124] Kerr J.P., Ziman A.P., Mueller A.L., Muriel J.M., Kleinhans-Welte E., Gumerson J.D., Vogel S.S., Ward C.W., Roche J.A., Bloch R.J. (2013). Dysferlin stabilizes stress-induced Ca^2+^ signaling in the transverse tubule membrane. Proc. Natl. Acad. Sci. USA.

[125] Demonbreun A.R., Rossi A.E., Alvarez M.G., Swanson K.E., Deveaux H.K., Earley J.U., Hadhazy M., Vohra R., Walter G.A., Pytel P. (2014). Dysferlin and myoferlin regulate transverse tubule formation and glycerol sensitivity. Am. J. Pathol..

[126] Ampong B.N., Imamura M., Matsumiya T., Yoshida M., Takeda S. (2005). Intracellular localization of dysferlin and its association with the dihydropyridine receptor. Acta Myol..

[127] DiFranco M., Neco P., Capote J., Meera P., Vergara J.L. (2006). Quantitative evaluation of mammalian skeletal muscle as a heterologous protein expression system. Protein Expr. Purif..

[128] DiFranco M., Quinonez M., Capote J., Vergara J. (2009). DNA transfection of mammalian skeletal muscles using in vivo electroporation. J. Vis. Exp..

[129] Hofhuis J., Bersch K., Büssenschütt R., Drzymalski M., Liebetanz D., Nikolaev V.O., Wagner S., Maier L.S., Gärtner J., Klinge L. (2017). Dysferlin mediates membrane tubulation and links T-tubule biogenesis to muscular dystrophy. J. Cell Sci..

[130] Launikonis B.S., Murphy R.M. (2025). From Muscle-Based Nonshivering Thermogenesis to Malignant Hyperthermia in Mammals. Annu. Rev. Physiol..

[131] Waddell L.B., Lemckert F.A., Zheng X.F., Tran J., Evesson F.J., Hawkes J.M., Lek A., Street N.E., Lin P., Clarke N.F. (2011). Dysferlin, annexin A1, and mitsugumin 53 are upregulated in muscular dystrophy and localize to longitudinal tubules of the T-system with stretch. J. Neuropathol. Exp. Neurol..

[132] Paulke N.J., Fleischhacker C., Wegener J.B., Riedemann G.C., Cretu C., Mushtaq M., Zaremba N., Möbius W., Zühlke Y., Wedemeyer J. (2024). Dysferlin enables tubular membrane proliferation in cardiac hypertrophy. Circ. Res..

[133] Franzini-Armstrong C., Protasi F., Ramesh V. (1999). Shape, size, and distribution of Ca^2+^ release units and couplons in skeletal and cardiac muscles. Biophys. J..

[134] Kerr J.P., Ward C.W., Bloch R.J. (2014). Dysferlin at transverse tubules regulates Ca^2+^ homeostasis in skeletal muscle. Front. Physiol..

[135] Ríos E., Figueroa L., Manno C., Kraeva N., Riazi S. (2015). The couplonopathies: A comparative approach to a class of diseases of skeletal and cardiac muscle. J. Gen. Physiol..

[136] Ríos E. (2018). Calcium-induced release of calcium in muscle: 50 years of work and the emerging consensus. J. Gen. Physiol..

[137] Xu J., Liao C., Yin C.C., Li G., Zhu Y., Sun F. (2024). In situ structural insights into the excitation-contraction coupling mechanism of skeletal muscle. Sci. Adv..

[138] Lukyanenko V., Muriel J.M., Bloch R.J. (2017). Coupling of excitation to Ca^2+^ release is modulated by dysferlin. J. Physiol..

[139] Lukyanenko V., Muriel J., Garman D., Breydo L., Bloch R.J. (2022). Elevated Ca^2+^ at the triad junction underlies dysregulation of Ca(2+) signaling in dysferlin-null skeletal muscle. Front. Physiol..

[140] Barefield D.Y., Sell J.J., Tahtah I., Kearns S.D., McNally E.M., Demonbreun A.R. (2021). Loss of dysferlin or myoferlin results in differential defects in excitation-contraction coupling in mouse skeletal muscle. Sci. Rep..

[141] Canato M., Capitanio P., Reggiani C., Cancellara L. (2015). The disorders of the calcium release unit of skeletal muscles: What have we learned from mouse models?. J. Muscle Res. Cell Motil..

[142] Rudolf R., Magalhães P.J., Pozzan T. (2006). Direct in vivo monitoring of sarcoplasmic reticulum Ca^2+^ and cytosolic cAMP dynamics in mouse skeletal muscle. J. Cell Biol..

[143] Sztretye M., Yi J., Figueroa L., Zhou J., Royer L., Ríos E. (2011). D4cpv-calsequestrin: A sensitive ratiometric biosensor accurately targeted to the calcium store of skeletal muscle. J. Gen. Physiol..

[144] Ziman A.P., Ward C.W., Rodney G.G., Lederer W.J., Bloch R.J. (2010). Quantitative measurement of Ca^2+^ in the sarcoplasmic reticulum lumen of mammalian skeletal muscle. Biophys. J..

[145] Meizoso-Huesca A., Lamboley C.R., Krycer J.R., Hodson M.P., Hudson J.E., Launikonis B.S. (2025). Muscle-specific Ryanodine receptor 1 properties underlie limb-girdle muscular dystrophy 2B/R2 progression. Nat. Commun..

[146] Isaeva E.V., Shkryl V.M., Shirokova N. (2005). Mitochondrial redox state and Ca^2+^ sparks in permeabilized mammalian skeletal muscle. J. Physiol..

[147] Kirsch W.G., Uttenweiler D., Fink R.H. (2001). Spark- and ember-like elementary Ca^2+^ release events in skinned fibres of adult mammalian skeletal muscle. J. Physiol..

[148] Uttenweiler D., Kirsch W.G., Schulzke E., Both M., Fink R.H. (2002). Model-based analysis of elementary Ca^2+^ release events in skinned mammalian skeletal muscle fibres. Eur. Biophys. J..

[149] Aydin J., Shabalina I.G., Place N., Reiken S., Zhang S.J., Bellinger A.M., Nedergaard J., Cannon B., Marks A.R., Bruton J.D. (2008). Nonshivering thermogenesis protects against defective calcium handling in muscle. FASEB J..

[150] Bal N.C., Periasamy M. (2020). Uncoupling of sarcoendoplasmic reticulum calcium ATPase pump activity by sarcolipin as the basis for muscle non-shivering thermogenesis. Philos. Trans. R. Soc. Lond. B Biol. Sci..

[151] Barclay C.J., Launikonis B.S. (2022). A mathematical model to quantify RYR Ca^2+^ leak and associated heat production in resting human skeletal muscle fibers. J. Gen. Physiol..

[152] Bruton J.D., Aydin J., Yamada T., Shabalina I.G., Ivarsson N., Zhang S.J., Wada M., Tavi P., Nedergaard J., Katz A. (2010). Increased fatigue resistance linked to Ca^2+^-stimulated mitochondrial biogenesis in muscle fibres of cold-acclimated mice. J. Physiol..

[153] Heemstra L.A., Koch L.G., Britton S.L., Novak C.M. (2022). Altered skeletal muscle sarco-endoplasmic reticulum Ca(^2+^)-ATPase calcium transport efficiency after a thermogenic stimulus. Am. J. Physiol. Regul. Integr. Comp. Physiol..

[154] Singh D.P., Pearce L., Choi R.H., Meizoso-Huesca A., Wette S.G., Scott J.W., Lamboley C.R., Murphy R.M., Launikonis B.S. (2023). Evolutionary isolation of ryanodine receptor isoform 1 for muscle-based thermogenesis in mammals. Proc. Natl. Acad. Sci. USA.

[155] Cully T.R., Choi R.H., Bjorksten A.R., Stephenson D.G., Murphy R.M., Launikonis B.S. (2018). Junctional membrane Ca^2+^ dynamics in human muscle fibers are altered by malignant hyperthermia causative RyR mutation. Proc. Natl. Acad. Sci. USA.

[156] Dirksen R.T., Avila G. (2004). Distinct effects on Ca^2+^ handling caused by malignant hyperthermia and central core disease mutations in RyR1. Biophys. J..

[157] Eltit J.M., Bannister R.A., Moua O., Altamirano F., Hopkins P.M., Pessah I.N., Molinski T.F., López J.R., Beam K.G., Allen P.D. (2012). Malignant hyperthermia susceptibility arising from altered resting coupling between the skeletal muscle L-type Ca^2+^ channel and the type 1 ryanodine receptor. Proc. Natl. Acad. Sci. USA.

[158] Lanner J.T., Georgiou D.K., Joshi A.D., Hamilton S.L. (2010). Ryanodine receptors: Structure, expression, molecular details, and function in calcium release. Cold Spring Harb. Perspect. Biol..

[159] Tong J., McCarthy T.V., MacLennan D.H. (1999). Measurement of resting cytosolic Ca^2+^ concentrations and Ca^2+^ store size in HEK-293 cells transfected with malignant hyperthermia or central core disease mutant Ca^2+^ release channels. J. Biol. Chem..

[160] Endo M. (2009). Calcium-induced calcium release in skeletal muscle. Physiol. Rev..

[161] Gehlert S., Bloch W., Suhr F. (2015). Ca^2+^-dependent regulations and signaling in skeletal muscle: From electro-mechanical coupling to adaptation. Int. J. Mol. Sci..

[162] Head S.I. (1993). Membrane potential, resting calcium and calcium transients in isolated muscle fibres from normal and dystrophic mice. J. Physiol..

[163] Williams D.A., Head S.I., Bakker A.J., Stephenson D.G. (1990). Resting calcium concentrations in isolated skeletal muscle fibres of dystrophic mice. J. Physiol..

[164] Csernoch L. (2007). Sparks and embers of skeletal muscle: The exciting events of contractile activation. Pflugers Arch..

[165] Iino M. (1989). Calcium-induced calcium release mechanism in guinea pig taenia caeci. J. Gen. Physiol..

[166] Rodríguez M.D., Morris J.A., Bardsley O.J., Matthews H.R., Huang C.L. (2024). Nernst-Planck-Gaussian finite element modelling of Ca^2+^ electrodiffusion in amphibian striated muscle transverse tubule-sarcoplasmic reticular triadic junctional domains. Front. Physiol..

[167] Takahashi Y., Furukawa K., Kozutsumi D., Ishibashi M., Kobayashi J., Ohizumi Y. (1995). 4,6-Dibromo-3-hydroxycarbazole (an analogue of caffeine-like Ca^2+^ releaser), a novel type of inhibitor of Ca^2+^-induced Ca^2+^ release in skeletal muscle sarcoplasmic reticulum. Br. J. Pharmacol..

[168] Fill M., Mejía-Alvarez R., Kettlun C., Escobar A. (1999). Ryanodine receptor permeation and gating: Glowing cinders that underlie the Ca^2+^ spark. J. Gen. Physiol..

[169] Fill M. (2003). Mechanisms that turn-off intracellular calcium release channels. Front. Biosci..

[170] Porta M., Diaz-Sylvester P.L., Neumann J.T., Escobar A.L., Fleischer S., Copello J.A. (2012). Coupled gating of skeletal muscle ryanodine receptors is modulated by Ca^2+^, Mg^2+^, and ATP. Am. J. Physiol. Cell Physiol..

[171] Stephenson D.G. (2024). Modeling the mechanism of Ca^2+^ release in skeletal muscle by DHPRs easing inhibition at RyR 1-sites. J. Gen. Physiol..

[172] Ivarsson N., Mattsson C.M., Cheng A.J., Bruton J.D., Ekblom B., Lanner J.T., Westerblad H. (2019). SR Ca^2+^ leak in skeletal muscle fibers acts as an intracellular signal to increase fatigue resistance. J. Gen. Physiol..

[173] Lamboley C.R., Pearce L., Seng C., Meizoso-Huesca A., Singh D.P., Frankish B.P., Kaura V., Lo H.P., Ferguson C., Allen P.D. (2021). Ryanodine receptor leak triggers fiber Ca^2+^ redistribution to preserve force and elevate basal metabolism in skeletal muscle. Sci. Adv..

[174] Kobayashi T., Yamazawa T., Kurebayashi N., Konishi M., Tanihata J., Sugihara M., Miki Y., Noguchi S., Inoue Y.U., Inoue T. (2025). RyR1-mediated Ca^2+^-induced Ca^2+^ release plays a negligible role in excitation-contraction coupling of normal skeletal muscle. Proc. Natl. Acad. Sci. USA.

[175] Iotti S., Frassineti C., Alderighi L., Sabatini A., Vacca A., Barbiroli B. (2000). In vivo (31)P-MRS assessment of cytosolic [Mg^2+^] in the human skeletal muscle in different metabolic conditions. Magn. Reson. Imaging.

[176] Donoso P., Aracena P., Hidalgo C. (2000). Sulfhydryl oxidation overrides Mg^2+^ inhibition of calcium-induced calcium release in skeletal muscle triads. Biophys. J..

[177] Csernoch L., Bernengo J.C., Szentesi P., Jacquemond V. (1998). Measurements of intracellular Mg^2+^ concentration in mouse skeletal muscle fibers with the fluorescent indicator mag-indo-1. Biophys. J..

[178] Jeacocke R.E. (1993). The concentrations of free magnesium and free calcium ions both increase in skeletal muscle fibres entering rigor mortis. Meat Sci..

[179] Allen D.G., Lännergren J., Westerblad H. (2002). Intracellular ATP measured with luciferin/luciferase in isolated single mouse skeletal muscle fibres. Pflugers Arch..

[180] Bennetts B., Parker M.W., Cromer B.A. (2007). Inhibition of skeletal muscle ClC-1 chloride channels by low intracellular pH and ATP. J. Biol. Chem..

[181] Stange M., Xu L., Balshaw D., Yamaguchi N., Meissner G. (2003). Characterization of recombinant skeletal muscle (Ser-2843) and cardiac muscle (Ser-2809) ryanodine receptor phosphorylation mutants. J. Biol. Chem..

[182] Bellinger A.M., Reiken S., Dura M., Murphy P.W., Deng S.X., Landry D.W., Nieman D., Lehnart S.E., Samaru M., LaCampagne A. (2008). Remodeling of ryanodine receptor complex causes “leaky” channels: A molecular mechanism for decreased exercise capacity. Proc. Natl. Acad. Sci. USA.

[183] Bellinger A.M., Reiken S., Carlson C., Mongillo M., Liu X., Rothman L., Matecki S., Lacampagne A., Marks A.R. (2009). Hypernitrosylated ryanodine receptor calcium release channels are leaky in dystrophic muscle. Nat. Med..

[184] Cully T.R., Rodney G.G. (2020). Nox4-RyR1-Nox2: Regulators of micro-domain signaling in skeletal muscle. Redox Biol..

[185] Durham W.J., Aracena-Parks P., Long C., Rossi A.E., Goonasekera S.A., Boncompagni S., Galvan D.L., Gilman C.P., Baker M.R., Shirokova N. (2008). RyR1 S-nitrosylation underlies environmental heat stroke and sudden death in Y522S RyR1 knockin mice. Cell.

[186] Lanner J.T., Georgiou D.K., Dagnino-Acosta A., Ainbinder A., Cheng Q., Joshi A.D., Chen Z., Yarotskyy V., Oakes J.M., Lee C.S. (2012). AICAR prevents heat-induced sudden death in RyR1 mutant mice independent of AMPK activation. Nat. Med..

[187] Regan J.N., Mikesell C., Reiken S., Xu H., Marks A.R., Mohammad K.S., Guise T.A., Waning D.L. (2017). Osteolytic breast cancer causes skeletal muscle weakness in an immunocompetent syngeneic mouse model. Front. Endocrinol..

[188] Steinz M.M., Beard N., Shorter E., Lanner J.T. (2024). Stable oxidative posttranslational modifications alter the gating properties of RyR1. J. Gen. Physiol..

[189] Yamada T., Steinz M.M., Kenne E., Lanner J.T. (2017). Muscle weakness in rheumatoid arthritis: The role of Ca(^2+^) and free radical signaling. eBioMedicine.

[190] Sun J., Xu L., Eu J.P., Stamler J.S., Meissner G. (2003). Nitric oxide, NOC-12, and S-nitrosoglutathione modulate the skeletal muscle calcium release channel/ryanodine receptor by different mechanisms. An allosteric function for O_2_ in S-nitrosylation of the channel. J. Biol. Chem..

[191] Place N., Ivarsson N., Venckunas T., Neyroud D., Brazaitis M., Cheng A.J., Ochala J., Kamandulis S., Girard S., Volungevičius G. (2015). Ryanodine receptor fragmentation and sarcoplasmic reticulum Ca^2+^ leak after one session of high-intensity interval exercise. Proc. Natl. Acad. Sci. USA.

[192] Henríquez-Olguín C., Díaz-Vegas A., Utreras-Mendoza Y., Campos C., Arias-Calderón M., Llanos P., Contreras-Ferrat A., Espinosa A., Altamirano F., Jaimovich E. (2016). NOX2 inhibition impairs early muscle gene expression induced by a single exercise bout. Front. Physiol..

[193] Henríquez-Olguín C., Boronat S., Cabello-Verrugio C., Jaimovich E., Hidalgo E., Jensen T.E. (2019). The emerging roles of nicotinamide adenine dinucleotide phosphate oxidase 2 in skeletal muscle redox signaling and metabolism. Antioxid. Redox Signal.

[194] Henriquez-Olguin C., Meneses-Valdes R., Raun S.H., Gallero S., Knudsen J.R., Li Z., Li J., Sylow L., Jaimovich E., Jensen T.E. (2023). NOX2 deficiency exacerbates diet-induced obesity and impairs molecular training adaptations in skeletal muscle. Redox Biol..

[195] Liu J., Zhou G., Mei Y., Xie W.J., Li P.F., Yang F. (2020). [Mechanism of oxidative stress in skeletal muscle of rats induced by acute exhaustive exercise]. Zhongguo Ying Yong Sheng Li Xue Za Zhi.

[196] Liu X.H., Harlow L., Graham Z.A., Bauman W.A., Cardozo C. (2017). spinal cord injury leads to hyperoxidation and nitrosylation of skeletal muscle ryanodine receptor-1 associated with upregulation of nicotinamide adenine dinucleotide phosphate oxidase 4. J. Neurotrauma.

[197] Osório Alves J., Pereira L.M., Monteiro I.C.C.D.R., Santos L.H.P.D., Ferraz A.S.M., Loureiro A.C.C., Lima C.C., Leal-Cardoso J.H., Carvalho D.P., Fortunato R.S. (2020). Strenuous acute exercise induces slow and fast twitch-dependent NADPH oxidase expression in rat skeletal muscle. Antioxidants.

[198] Prosser B.L., Khairallah R.J., Ziman A.P., Ward C.W., Lederer W.J. (2013). X-ROS signaling in the heart and skeletal muscle: Stretch-dependent local ROS regulates [Ca^2+^]_i_. J. Mol. Cell Cardiol..

[199] Specht K.S., Kant S., Addington A.K., McMillan R.P., Hulver M.W., Learnard H., Campbell M., Donnelly S.R., Caliz A.D., Pei Y. (2021). Nox4 mediates skeletal muscle metabolic responses to exercise. Mol. Metab..

[200] Wang D., Jiang D.M., Yu R.R., Zhang L.L., Liu Y.Z., Chen J.X., Chen H.C., Liu Y.P. (2022). The effect of aerobic exercise on the oxidative capacity of skeletal muscle mitochondria in mice with impaired glucose tolerance. J. Diabetes Res..

[201] Ward C.W., Prosser B.L., Lederer W.J. (2014). Mechanical stretch-induced activation of ROS/RNS signaling in striated muscle. Antioxid. Redox Signal.

[202] Xirouchaki C.E., Jia Y., McGrath M.J., Greatorex S., Tran M., Merry T.L., Hong D., Eramo M.J., Broome S.C., Woodhead J.S.T. (2021). Skeletal muscle NOX4 is required for adaptive responses that prevent insulin resistance. Sci. Adv..

[203] Gehlert S., Bungartz G., Willkomm L., Korkmaz Y., Pfannkuche K., Schiffer T., Bloch W., Suhr F. (2012). Intense resistance exercise induces early and transient increases in ryanodine receptor 1 phosphorylation in human skeletal muscle. PLoS ONE.

[204] Jacko D., Bersiner K., Friederichs G., Ritter P., Nirenberg L., Eisenbraun J., de Marées M., Bloch W., Gehlert S. (2018). Resistance exercise-induced muscle fatigue is not accompanied by increased phosphorylation of ryanodine receptor 1 at serine 2843. PLoS ONE.

[205] Dridi H., Wu W., Reiken S.R., Ofer R.M., Liu Y., Yuan Q., Sittenfeld L., Kushner J., Muchir A., Worman H.J. (2021). Ryanodine receptor remodeling in cardiomyopathy and muscular dystrophy caused by lamin A/C gene mutation. Hum. Mol. Genet..

[206] Lotteau S., Ivarsson N., Yang Z., Restagno D., Colyer J., Hopkins P., Weightman A., Himori K., Yamada T., Bruton J. (2019). A mechanism for statin-induced susceptibility to myopathy. JACC Basic Transl. Sci..

[207] Matecki S., Dridi H., Jung B., Saint N., Reiken S.R., Scheuermann V., Mrozek S., Santulli G., Umanskaya A., Petrof B.J. (2016). Leaky ryanodine receptors contribute to diaphragmatic weakness during mechanical ventilation. Proc. Natl. Acad. Sci. USA.

[208] Reiken S., Lacampagne A., Zhou H., Kherani A., Lehnart S.E., Ward C., Huang F., Gaburjakova M., Gaburjakova J., Rosemblit N. (2003). PKA phosphorylation activates the calcium release channel (ryanodine receptor) in skeletal muscle: Defective regulation in heart failure. J. Cell Biol..

[209] Rullman E., Andersson D.C., Melin M., Reiken S., Mancini D.M., Marks A.R., Lund L.H., Gustafsson T. (2013). Modifications of skeletal muscle ryanodine receptor type 1 and exercise intolerance in heart failure. J. Heart Lung Transplant..

[210] Martuscello R.T., Chen M.L., Reiken S., Sittenfeld L.R., Ruff D.S., Ni C.L., Lin C.C., Pan M.K., Louis E.D., Marks A.R. (2023). Defective cerebellar ryanodine receptor type 1 and endoplasmic reticulum calcium ‘leak’ in tremor pathophysiology. Acta Neuropathol..

[211] Godbout K., Dugas M., Reiken S.R., Ramezani S., Falle A., Rousseau J., Wronska A.E., Lamothe G., Canet G., Lu Y. (2025). Universal prime editing therapeutic strategy for RyR1-related myopathies: A protective mutation rescues leaky RyR1 channel. Int. J. Mol. Sci..

[212] Ji L.L., Fu R., Mitchell E.W. (1992). Glutathione and antioxidant enzymes in skeletal muscle: Effects of fiber type and exercise intensity. J. Appl. Physiol..

[213] Kotidis E., Papavramidis T., Ioannidis K., Koliakos G., Lazou T., Cheva A., Michalopoulos N., Papavramidis S. (2012). Can chronic intra-abdominal hypertension cause oxidative stress to the abdominal wall muscles? An experimental study. J. Surg. Res..

[214] Leeuwenburgh C., Fiebig R., Chandwaney R., Ji L.L. (1994). Aging and exercise training in skeletal muscle: Responses of glutathione and antioxidant enzyme systems. Am. J. Physiol..

[215] Levine S., Nguyen T., Taylor N., Friscia M.E., Budak M.T., Rothenberg P., Zhu J., Sachdeva R., Sonnad S., Kaiser L.R. (2008). Rapid disuse atrophy of diaphragm fibers in mechanically ventilated humans. N. Engl. J. Med..

[216] Renjini R., Gayathri N., Nalini A., Bharath M.M.S. (2012). Oxidative damage in muscular dystrophy correlates with the severity of the pathology: Role of glutathione metabolism. Neurochem. Res..

[217] Bacchi M., Jullian M., Sirigu S., Fould B., Huet T., Bruyand L., Antoine M., Vuillard L., Ronga L., Chavas L.M. (2016). Total chemical synthesis, refolding, and crystallographic structure of fully active immunophilin calstabin 2 (FKBP12.6). Protein Sci..

[218] Gaburjakova M., Gaburjakova J., Reiken S., Huang F., Marx S.O., Rosemblit N., Marks A.R. (2001). FKBP12 binding modulates ryanodine receptor channel gating. J. Biol. Chem..

[219] Nakazawa T., Takasawa S., Noguchi N., Nata K., Tohgo A., Mori M., Nakagawara K., Akiyama T., Ikeda T., Yamauchi A. (2005). Genomic organization, chromosomal localization, and promoter of human gene for FK506-binding protein 12.6. Gene.

[220] Andersson D.C., Marks A.R. (2010). Fixing ryanodine receptor Ca leak—A novel therapeutic strategy for contractile failure in heart and skeletal muscle. Drug Discov. Today Dis. Mech..

[221] Lehnart S.E. (2007). Novel targets for treating heart and muscle disease: Stabilizing ryanodine receptors and preventing intracellular calcium leak. Curr. Opin. Pharmacol..

[222] Wehrens X.H., Lehnart S.E., Marks A.R. (2005). Intracellular calcium release and cardiac disease. Annu. Rev. Physiol..

[223] Protasi F., Franzini-Armstrong C., Flucher B.E. (2007). Coordinated incorporation of muscle dihydropyridine receptors and ryanodine receptors in peripheral couplings of BC3H1 cells. J. Cell Biol..

[224] Lawal T.A., Todd J.J., Witherspoon J.W., Bönnemann C.G., Dowling J.J., Hamilton S.L., Meilleur K.G., Dirksen R.T. (2020). Ryanodine receptor 1-related disorders: An historical perspective and proposal for a unified nomenclature. Skelet. Muscle.

[225] MacLennan D.H. (2000). Ca^2+^ signalling and muscle disease. Eur. J. Biochem..

[226] Riazi S., Kraeva N., Hopkins P.M. (2018). Updated guide for the management of malignant hyperthermia. Can. J. Anaesth..

[227] Yang H.S., Choi J.M., In J., Sung T.Y., Kim Y.B., Sultana S. (2023). Current clinical application of dantrolene sodium. Anesth. Pain Med..

[228] Flachenecker P., Kiefer R., Naumann M., Handwerker M., Reichmann H. (1997). Distal muscular dystrophy of Miyoshi type. Report of two cases and review of the literature. J. Neurol..

[229] Hanisch F., Kronenberger C., Zierz S., Kornhuber M. (2014). The significance of pathological spontaneous activity in various myopathies. Clin. Neurophysiol..

[230] Park K.H., Weisleder N., Zhou J., Gumpper K., Zhou X., Duann P., Ma J., Lin P.H. (2014). Assessment of calcium sparks in intact skeletal muscle fibers. J. Vis. Exp..

[231] Shkryl V.M., Martins A.S., Ullrich N.D., Nowycky M.C., Niggli E., Shirokova N. (2009). Reciprocal amplification of ROS and Ca^2+^ signals in stressed mdx dystrophic skeletal muscle fibers. Pflugers Arch..

[232] Teichmann M.D., Wegner F.V., Fink R.H., Chamberlain J.S., Launikonis B.S., Martinac B., Friedrich O. (2008). Inhibitory control over Ca^2+^ sparks via mechanosensitive channels is disrupted in dystrophin deficient muscle but restored by mini-dystrophin expression. PLoS ONE.

[233] Wang X., Weisleder N., Collet C., Zhou J., Chu Y., Hirata Y., Zhao X., Pan Z., Brotto M., Cheng H. (2005). Uncontrolled calcium sparks act as a dystrophic signal for mammalian skeletal muscle. Nat. Cell Biol..

[234] Weisleder N., Ma J.J. (2006). Ca^2+^ sparks as a plastic signal for skeletal muscle health, aging, and dystrophy. Acta Pharmacol. Sin..

[235] Cheng H., Lederer W.J., Cannell M.B. (1993). Calcium sparks: Elementary events underlying excitation-contraction coupling in heart muscle. Science.

[236] Cserne Szappanos H., Vincze J., Bodnár D., Dienes B., Schneider M.F., Csernoch L., Szentesi P. (2020). High Time Resolution Analysis of Voltage-Dependent and Voltage-Independent Calcium Sparks in Frog Skeletal Muscle Fibers. Front. Physiol..

[237] Lacampagne A., Lederer W.J., Schneider M.F., Klein M.G. (1996). Repriming and activation alter the frequency of stereotyped discrete Ca^2+^ release events in frog skeletal muscle. J. Physiol..

[238] Stern M.D., Pizarro G., Ríos E. (1997). Local control model of excitation-contraction coupling in skeletal muscle. J. Gen. Physiol..

[239] Kutchukian C., Szentesi P., Allard B., Buj-Bello A., Csernoch L., Jacquemond V. (2019). Ca^2+^-induced sarcoplasmic reticulum Ca^2+^ release in myotubularin-deficient muscle fibers. Cell Calcium.

[240] Tjondrokoesoemo A., Park K.H., Ferrante C., Komazaki S., Lesniak S., Brotto M., Ko J.K., Zhou J., Weisleder N., Ma J. (2011). Disrupted membrane structure and intracellular Ca^2+^ signaling in adult skeletal muscle with acute knockdown of Bin1. PLoS ONE.

[241] Vincze J., Jenes Á., Füzi M., Almássy J., Németh R., Szigeti G., Dienes B., Gaál Z., Szentesi P., Jóna I. (2015). Effects of fluvastatin and coenzyme Q10 on skeletal muscle in normo- and hypercholesterolaemic rats. J. Muscle Res. Cell Motil..

[242] Kushnir A., Wajsberg B., Marks A.R. (2018). Ryanodine receptor dysfunction in human disorders. Biochim. Biophys. Acta Mol. Cell Res..

[243] Calderón J.C., Bolaños P., Caputo C. (2014). Tetanic Ca^2+^ transient differences between slow- and fast-twitch mouse skeletal muscle fibres: A comprehensive experimental approach. J. Muscle Res. Cell Motil..

[244] Carroll S., Nicotera P., Pette D. (1999). Calcium transients in single fibers of low-frequency stimulated fast-twitch muscle of rat. Am. J. Physiol..

[245] Eisner D., Neher E., Taschenberger H., Smith G. (2023). Physiology of intracellular calcium buffering. Physiol. Rev..

[246] Manno C., Sztretye M., Figueroa L., Allen P.D., Ríos E. (2013). Dynamic measurement of the calcium buffering properties of the sarcoplasmic reticulum in mouse skeletal muscle. J. Physiol..

[247] Eshima H., Miura S., Senoo N., Hatakeyama K., Poole D.C., Kano Y. (2017). Improved skeletal muscle Ca^2+^ regulation in vivo following contractions in mice overexpressing PGC-1α. Am. J. Physiol. Regul. Integr. Comp. Physiol..

[248] Marcucci L., Nogara L., Canato M., Germinario E., Raffaello A., Carraro M., Bernardi P., Pietrangelo L., Boncompagni S., Protasi F. (2024). Mitochondria can substitute for parvalbumin to lower cytosolic calcium levels in the murine fast skeletal muscle. Acta Physiol..

[249] Murphy R.M., Larkins N.T., Mollica J.P., Beard N.A., Lamb G.D. (2009). Calsequestrin content and SERCA determine normal and maximal Ca^2+^ storage levels in sarcoplasmic reticulum of fast- and slow-twitch fibres of rat. J. Physiol..

[250] Royer L., Sztretye M., Manno C., Pouvreau S., Zhou J., Knollmann B.C., Protasi F., Allen P.D., Ríos E. (2010). Paradoxical buffering of calcium by calsequestrin demonstrated for the calcium store of skeletal muscle. J. Gen. Physiol..

[251] Helassa N., Podor B., Fine A., Török K. (2016). Design and mechanistic insight into ultrafast calcium indicators for monitoring intracellular calcium dynamics. Sci. Rep..

[252] Galbiati F., Volonte D., Minetti C., Chu J.B., Lisanti M.P. (1999). Phenotypic behavior of caveolin-3 mutations that cause autosomal dominant limb girdle muscular dystrophy (LGMD-1C). Retention of LGMD-1C caveolin-3 mutants within the golgi complex. J. Biol. Chem..

[253] Khan A.H., Capilla E., Hou J.C., Watson R.T., Smith J.R., Pessin J.E. (2004). Entry of newly synthesized GLUT4 into the insulin-responsive storage compartment is dependent upon both the amino terminus and the large cytoplasmic loop. J. Biol. Chem..

[254] Luetterforst R., Stang E., Zorzi N., Carozzi A., Way M., Parton R.G. (1999). Molecular characterization of caveolin association with the Golgi complex: Identification of a cis-Golgi targeting domain in the caveolin molecule. J. Cell Biol..

[255] Ploug T., van Deurs B., Ai H., Cushman S.W., Ralston E. (1998). Analysis of GLUT4 distribution in whole skeletal muscle fibers: Identification of distinct storage compartments that are recruited by insulin and muscle contractions. J. Cell Biol..

[256] Rahkila P., Alakangas A., Väänänen K., Metsikkö K. (1996). Transport pathway, maturation, and targetting of the vesicular stomatitis virus glycoprotein in skeletal muscle fibers. J. Cell Sci..

[257] Tajika Y., Takahashi M., Khairani A.F., Ueno H., Murakami T., Yorifuji H. (2014). Vesicular transport system in myotubes: Ultrastructural study and signposting with vesicle-associated membrane proteins. Histochem. Cell Biol..

[258] Flix B., de la Torre C., Castillo J., Casal C., Illa I., Gallardo E. (2013). Dysferlin interacts with calsequestrin-1, myomesin-2 and dynein in human skeletal muscle. Int. J. Biochem. Cell Biol..

[259] Huang Y., Laval S.H., van Remoortere A., Baudier J., Benaud C., Anderson L.V., Straub V., Deelder A., Frants R.R., den Dunnen J.T. (2007). AHNAK, a novel component of the dysferlin protein complex, redistributes to the cytoplasm with dysferlin during skeletal muscle regeneration. FASEB J..

[260] Huang Y., de Morrée A., van Remoortere A., Bushby K., Frants R.R., den Dunnen J.T., van der Maarel S.M. (2008). Calpain 3 is a modulator of the dysferlin protein complex in skeletal muscle. Hum. Mol. Genet..

[261] Seror P., Krahn M., Laforet P., Leturcq F., Maisonobe T. (2008). Complete fatty degeneration of lumbar erector spinae muscles caused by a primary dysferlinopathy. Muscle Nerve.

[262] Costelli P., Reffo P., Penna F., Autelli R., Bonelli G., Baccino F.M. (2005). Ca^2+^-dependent proteolysis in muscle wasting. Int. J. Biochem. Cell Biol..

[263] Dupont-Versteegden E.E. (2005). Apoptosis in muscle atrophy: Relevance to sarcopenia. Exp. Gerontol..

[264] Huang J., Zhu X. (2016). The molecular mechanisms of calpains action on skeletal muscle atrophy. Physiol. Res..

[265] Powers S.K., Ozdemir M., Hyatt H. (2020). Redox control of proteolysis during inactivity-induced skeletal muscle atrophy. Antioxid. Redox Signal.

[266] Wing S.S., Lecker S.H., Jagoe R.T. (2011). Proteolysis in illness-associated skeletal muscle atrophy: From pathways to networks. Crit. Rev. Clin. Lab. Sci..

[267] Chin E.R. (2005). Role of Ca^2+^/calmodulin-dependent kinases in skeletal muscle plasticity. J. Appl. Physiol..

[268] Tavi P., Westerblad H. (2011). The role of in vivo Ca^2+^ signals acting on Ca^2+^-calmodulin-dependent proteins for skeletal muscle plasticity. J. Physiol..

[269] Al-Shanti N., Stewart C.E. (2009). Ca^2+^/calmodulin-dependent transcriptional pathways: Potential mediators of skeletal muscle growth and development. Biol. Rev. Camb. Philos. Soc..

[270] Ojuka E.O., Goyaram V., Smith J.A. (2012). The role of CaMKII in regulating GLUT4 expression in skeletal muscle. Am. J. Physiol. Endocrinol. Metab..

[271] Ahn B., Ranjit R., Premkumar P., Pharaoh G., Piekarz K.M., Matsuzaki S., Claflin D.R., Riddle K., Judge J., Bhaskaran S. (2019). Mitochondrial oxidative stress impairs contractile function but paradoxically increases muscle mass via fibre branching. J. Cachexia Sarcopenia Muscle.

[272] Cheng A.J., Bruton J.D., Lanner J.T., Westerblad H. (2015). Antioxidant treatments do not improve force recovery after fatiguing stimulation of mouse skeletal muscle fibres. J. Physiol..

[273] Espinosa A., Casas M., Jaimovich E. (2023). Energy (and reactive oxygen species generation) saving distribution of mitochondria for the activation of ATP production in skeletal muscle. Antioxidants.

[274] Dirksen R.T. (2009). Sarcoplasmic reticulum-mitochondrial through-space coupling in skeletal muscle. Appl. Physiol. Nutr. Metab..

[275] Alderton J.M., Steinhardt R.A. (2000). How calcium influx through calcium leak channels is responsible for the elevated levels of calcium-dependent proteolysis in dystrophic myotubes. Cardiovasc. Med..

[276] Andersson D.C., Meli A.C., Reiken S., Betzenhauser M.J., Umanskaya A., Shiomi T., D’Armiento J., Marks A.R. (2012). Leaky ryanodine receptors in beta-sarcoglycan deficient mice: A potential common defect in muscular dystrophy. Skelet Muscle.

[277] Brinkmeier H. (2011). TRP channels in skeletal muscle: Gene expression, function and implications for disease. Adv. Exp. Med. Biol..

[278] Bround M.J., Abay E., Huo J., Havens J.R., York A.J., Bers D.M., Molkentin J.D. (2024). MCU-in dependent Ca^2+^ uptake mediates mitochondrial Ca^2+^ overload and necrotic cell death in a mouse model of Duchenne muscular dystrophy. Sci. Rep..

[279] Burr A.R., Molkentin J.D. (2015). Genetic evidence in the mouse solidifies the calcium hypothesis of myofiber death in muscular dystrophy. Cell Death Differ..

[280] Hopf F.W., Turner P.R., Stainhardt R.A. (2007). Calcium misregulation and the pathogenesis of muscular dystrophy. Subcell Biochem..

[281] Kramerova I., Kudryashova E., Wu B., Ottenheijm C., Granzier H., Spencer M.J. (2008). Novel role of calpain-3 in the triad-associated protein complex regulating calcium release in skeletal muscle. Hum. Mol. Genet..

[282] Lindsay A., Baumann C.W., Rebbeck R.T., Yuen S.L., Southern W.M., Hodges J.S., Cornea R.L., Thomas D.D., Ervasti J.M., Lowe D.A. (2020). Mechanical factors tune the sensitivity of mdx muscle to eccentric strength loss and its protection by antioxidant and calcium modulators. Skelet Muscle.

[283] Marks A.R. (2023). Targeting ryanodine receptors to treat hyuman diseases. J. Clin. Investig..

[284] Grose W.E., Clark K.R., Griffin D., Malik V., Shontz K.M., Montgomery C.L., Lewis S., Brown R.H., Janssen P.M., Mendell J.R. (2012). Homologous recombination mediates functional recovery of dysferlin deficiency following AAV5 gene transfer. PLoS ONE.

[285] Potter R.A., Griffin D.A., Sondergaard P.C., Johnson R.W., Pozsgai E.R., Heller K.N., Peterson E.L., Lehtimäki K.K., Windish H.P., Mittal P.J. (2018). Systemic delivery of dysferlin overlap vectors provides long-term gene expression and functional improvement for dysferlinopathy. Hum. Gene Ther..

[286] Pryadkina M., Lostal W., Bourg N., Charton K., Roudaut C., Hirsch M.L., Richard I. (2015). A comparison of AAV strategies distinguishes overlapping vectors for efficient systemic delivery of the 6.2 kb Dysferlin coding sequence. Mol. Ther. Methods Clin. Dev..

[287] Sondergaard P.C., Griffin D.A., Pozsgai E.R., Johnson R.W., Grose W.E., Heller K.N., Shontz K.M., Montgomery C.L., Liu J., Clark K.R. (2015). AAV.Dysferlin overlap vectors restore function in dysferlinopathy animal models. Ann. Clin. Transl. Neurol..

